# Qihuang Zhuyu Formula Attenuates Atherosclerosis via Targeting PPAR*γ* to Regulate Cholesterol Efflux and Endothelial Cell Inflammation

**DOI:** 10.1155/2022/2226168

**Published:** 2022-12-05

**Authors:** Mengxi Wang, Qian Xiang, Weixin Sun, Haowen Zhang, Ruijie Shi, Jun Guo, Huaqin Tong, Manlu Fan, Yuhan Ding, Haibo Shi, Peng Yu, Le Shen, Qiong Wang, Xiaohu Chen

**Affiliations:** ^1^Department of Cardiology, Affiliated Hospital of Nanjing University of Chinese Medicine, Nanjing 210029, China; ^2^Department of Cardiology, Jiangsu Province Hospital of Chinese Medicine, Nanjing 210029, China; ^3^First Clinical Medical College, Nanjing University of Chinese Medicine, Nanjing 210023, China; ^4^Department of Cardiology, Yancheng TCM Hospital Affiliated to Nanjing University of Chinese Medicine, Yancheng 224000, China; ^5^College of Health Preservation and Rehabilitation, Nanjing University of Chinese Medicine, Nanjing 210023, China; ^6^Laboratory of Pharmacology, Affiliated Hospital of Nanjing University of Chinese Medicine, Nanjing 210029, China; ^7^Laboratory of Pharmacology, Jiangsu Province Hospital of Chinese Medicine, 210029 Nanjing, China

## Abstract

At present, due to the limitations of drug therapy targets for atherosclerosis, some patients fail to achieve satisfactory efficacy. Cholesterol efflux dysfunction and endothelial cell inflammation are considered to be important factors in the development of atherosclerosis. Peroxisome proliferator-activated receptor gamma (PPAR*γ*), a promising therapeutic target for atherosclerosis, plays a dual role in regulating cholesterol efflux and endothelial cell inflammation. However, the use of PPAR*γ* agonist in clinical practice is greatly limited as it could lead to water and sodium retention and hence result in congestive heart failure. Qihuang Zhuyu Formula (QHZYF) is a hospital preparation of Jiangsu Province Hospital of Chinese Medicine which has definite effect in the treatment of atherosclerosis, but its pharmacological mechanism has not been clear. In this study, we successfully predicted that QHZYF might regulate cholesterol efflux and endothelial inflammation via targeting PPAR*γ*-mediated PPAR*γ*/LXR*α*/ABCA1-ABCG1 and PPAR*γ*/NF-*κ*B p65 pathways by using UPLC-Q-TOF/MS, network pharmacology, bioinformatics analysis, and molecular docking technology. Subsequently, we confirmed in vivo that QHZYF could attenuate atherosclerosis in ApoE^−/−^ mice and regulate the expression levels of related molecules in PPAR*γ*/LXR*α*/ABCA1-ABCG1 and PPAR*γ*/NF-*κ*B p65 pathways of ApoE^−/−^ mice and C57BL/6 wild-type mice. Finally, in in vitro experiments, we found that QHZYF could reduce lipid content and increase cholesterol efflux rate of RAW 264.7 cells, inhibit the inflammatory response of HUVECs, and regulate the expression levels of related molecules in the two pathways. In addition, the above effects of QHZYF were significantly weakened after PPAR*γ* knockdown in the two kinds of cells. In conclusion, our study revealed that QHZYF attenuates atherosclerosis via targeting PPAR*γ*-mediated PPAR*γ*/LXR*α*/ABCA1-ABCG1 and PPAR*γ*/NF-*κ*B p65 pathways to regulate cholesterol efflux and endothelial cell inflammation. More importantly, our study offers a promising complementary and alternative therapy which is expected to make up for the limitation of current drug treatment methods and provide a valuable reference for new drugs development in the future.

## 1. Introduction

Atherosclerosis is a chronic disease characterized by the gradual accumulation of lipid and inflammatory cells in the endarterium and the formation of atherosclerotic plaques, resulting in thickening of the vascular wall and narrowing of the lumen [1]. Atherosclerotic cardiovascular disease is one of the leading causes of death and disability worldwide today [2]. The occurrence and development of atherosclerosis involve many kinds of pathogenesis, such as endothelial injury, hemodynamic changes, inflammatory infiltration, smooth muscle cell proliferation, and abnormal cholesterol metabolism [3]. At present, the pharmacological mechanism of the three main drugs for the treatment of atherosclerosis, 3-hydroxy-3-methyl glutaryl coenzyme A (HMG-CoA) reductase inhibitors, Niemann-pick C1-like protein 1 (NPC1L1) inhibitors, and proprotein convertase subtilisin/kexin type 9 (PCSK9) inhibitors, is mainly to reduce cholesterol through affecting the synthesis, absorption, and degradation of cholesterol, respectively. Although these drugs have achieved some positive therapeutic effects, a considerable number of patients still failed to benefit from these treatments, which prompted scholars to investigate other intervention targets for atherosclerosis [4].

Cholesterol efflux dysfunction and endothelial cell inflammation are considered to be key links in the occurrence and development of atherosclerosis independent of cholesterol synthesis, absorption, and degradation and have attracted considerable attention in recent years [5, 6]. The formation of foam cells under the intima is one of the main characteristics of atherosclerosis. Endothelial inflammation and cholesterol efflux, respectively, affect the early stage of foam cell formation and the terminal stage of foam cell removal. Stimulated by inflammation, endothelial cells may cause monocyte adhesion and migration to the subintima, which is the initial process of foam cell formation. After foam cell formation, cholesterol efflux is an important way for cells to excrete excess cholesterol and delay the progression of atherosclerosis. In the early stages of disease, endothelial cells are activated when stimulated by inflammatory factors, leading to cell dysfunction and increasing expression of various adhesion molecules such as intercellular cell adhesion molecule-1 (ICAM-1) and vascular endothelial cell adhesion molecule-1 (VCAM-1). Then, monocytes adhere to endothelial cells and migrate into the intima to phagocytose oxidized low-density lipoprotein (ox-LDL) to form foam cells, resulting in early atherosclerosis formation [7]. Later, with the continuous progression of the disease, a large amount of cholesterol is deposited in the lower layer of arterial intima. At that time, the body mainly relies on the cholesterol reversal transport (RCT) pathway to transfer the cholesterol in the cells of the extrahepatic tissues to the liver in the form of circulating high-density lipoprotein (HDL) particles, which is discharged in the form of feces after metabolism [8]. Cholesterol efflux is the first step and rate-limiting step of RCT, which is the key to affect the function of RCT [9]. Previous research has shown that cholesterol efflux efficiency has a more significant effect on atherosclerosis than HDL level [10]. Therefore, targeted regulation of cholesterol efflux and endothelial cell inflammation to treat atherosclerosis is a promising therapeutic direction, which is expected to make up for the deficiency of current drug therapy measures. However, there is still a lack of effective intervention methods in modern medicine. Therefore, further investigation is needed to explore relevant complementary and alternative therapies.

Traditional Chinese medicine (TCM) is one of the most important components of complementary and alternative therapies. It is characterized by multicomponents, multitargets, and overall regulation in the treatment of diseases. As is known to all, atherosclerosis is caused by the interaction of various pathological processes. Therefore, compared with modern medicine, TCM may have unique advantages in the coordinated treatment of atherosclerosis through multiple approaches. In recent years, more and more studies have found that the natural compounds contained in Chinese herbs have great potentials in regulating cholesterol efflux function and inhibiting endothelial cell inflammation, which has broad clinical application prospects [11, 12]. Further research on the active components and potential targets of Chinese herbs is conducive to providing novel ideas for the treatment of atherosclerosis and giving full play to the advantages of TCM.

The Qihuang Zhuyu Formula (QHZYF) is developed according to the theory of qi and blood of TCM. It is a hospital preparation of Jiangsu Province Hospital of Chinese Medicine and has applied for a national invention patent. It is composed of 5 kinds of herbs, including Milkvetch Root (*Astragali Radix*), Manyflower Solomonseal Rhizome (*Polygonati Rhizoma*), Sappan Wood (*Sappan Lignum*), Safflower (*Carthami Flos*), and Leech (*Hirudo*). The QHZYF has the effects of *Yiqi Huoxue* and *Huayu Tongmai*. It has been clinically used for many years and has definite efficacy in the treatment of atherosclerosis. However, it is still unclear about the pharmacological mechanism of QHZYF in the treatment of atherosclerosis, which hinders the further promotion of this prescription. Therefore, the present study intends to systematically investigate the effective ingredients and targets of QHZYF for the treatment of atherosclerosis with the help of UPLC-Q-TOF/MS, network pharmacology, bioinformatics analysis, and molecular docking technology. The results showed that peroxisome proliferator-activated receptor gamma- (PPAR*γ*-) mediated cholesterol efflux and endothelial cell inflammation may be the main targets of this prescription. After that, we further verified this hypothesis in in vivo and vitro experiments to provide reliable evidence for TCM treatment of atherosclerosis. More importantly, this study will help to remedy the limitations of current drug therapy measures and provide valuable reference for the development of new drugs for atherosclerosis in the future.

## 2. Materials and Methods

### 2.1. Preparation of QHZYF

The QHZYF is composed of Milkvetch Root (*Astragali Radix*), Manyflower So1omonseal Rhizome (*Polygonati Rhizoma*), Sappan Wood (*Sappan Lignum*), Safflower (*Carthami Flos*), and Leech (*Hirudo*) in a ratio of 30 : 15: 10: 10: 6. All the herbs were purchased from the Affiliated Hospital of Nanjing University of Chinese Medicine (Jiangsu Provincial Hospital of Chinese Medicine) and identified by two experienced traditional Chinese pharmacists. Firstly, the 5 kinds of herbs were mixed in the above proportion, soaked in 2 litres of double distilled water for 30 minutes, and boiled for 30 minutes. The liquid was then collected. Subsequently, another 2 litres of double distilled water was added into the herbs and boiled for another 30 minutes. After that, the obtained liquid was mixed and concentrated under reduced pressure (pressure controlled at -0.04-0.01 MPa, temperature controlled at about 60°C) to the relative density of about 1.05 and centrifuged by straight tube high-speed centrifuge (speed: 6000 r/min, flow rate: 4-6 L/min). Finally, the supernatant after centrifugation was taken to obtain the final preparation of QHZYF.

### 2.2. UPLC-Q-TOF/MS

1 mL of the prepared QHZYF (1 g/mL) was dissolved in 10 mL ultrapure water and mixed by vortex for 1 minute, followed by ice water bath ultrasound for 10 minutes. After that, the triploid volume of methanol solution was added to the sample. Then, they were mixed by vortex again for 1 minute and centrifugalized at 13000 rpm for 15 minutes. The supernatant was taken for UPLC-Q-TOF/MS analysis. In the processes, the instruments used include Waters Acquity UPLC Class I and Waters SYNAPT G2-S Q-TOF/MS High-Resolution MS (Waters Corporation, USA). Chromatographic separation was performed using Waters Acquity UPLC BEH C18 column (2.1 mm × 100 mm, 1.7 *μ*m) at a flow rate of 0.3 mL/min. The column temperature was 40°C, and sample size was 5 *μ*L each time. Mobile phase A was 0.1% formic acid aqueous solution, and mobile phase B was 0.1% formic acid acetonitrile. The gradient elution condition is set as follows: 0-9 min, 95-60% A; 9-19 min, 60-10% A; 19-25 min, 10-5% A; 25-27 min, 5% A; 27-28 min, 5-95% A; and 28-30 min, 95% A. Electrospray ionization source was used for analysis of mass spectrometry in positive and negative ion modes. The specific parameters were set as follows: high purity nitrogen was used as auxiliary spray and desolvent gas; dry gas flow rate, 10 mL/min; nitrogen temperature, 120°C; atomizing gas pressure, 310 kPa; desolvent nitrogen flow rate, 900 L/h; tapered hole reverse blowing flow rate, 50 L/h; capillary ionization voltage, 500 V; tapered hole voltage, 40 V; impact energy, 40-65 eV; scan range, 50-1500 Da; and mass range, m/z 50-1200.

### 2.3. Network Pharmacology and Bioinformatics Analysis

The TCMSP database (https://old.tcmsp-e.com/tcmsp.php) and BATMAN database (http://bionet.ncpsb.org.cn/batman-tcm/) were used to predict the targets of the effective components of QHZYF obtained by UPLC-Q-TOF/MS analysis, and the names of the obtained targets were standardized according to the UniProt database (http://www.uniprot.org/). The key words of “atherosclerosis” were used to search for disease-related therapeutic targets in the DrugBank database (https://www.drugbank.ca/), GeneCards database (https://www.genecards.or), OMIM database (https://omim.org/), TTD database (https://db.idrblab.org/ttd/), and PharmGKB database (https://www.pharmgkb.org/). The screening results were summarized, and duplicated genes were removed to obtain the common target of the active ingredient and atherosclerosis. Cytoscape 3.8.2 was used to construct the visualization network diagram of “drug-component-target.” The protein-protein interaction (PPI) network was constructed using the STRING database (https://string-db.org/). The exported file was imported into Cytoscape, and the network centrality analysis was performed twice using the CytoNCA plug-in to obtain the network core targets. Lastly, GO and KEGG enrichment analyses were performed, and bubble charts were drawn for the top 30 biological processes and signaling pathways. These procedures were implemented with packages “http://org.Hs.eg.db,” “colorspace,” “stringi,” “ggplot2,” “BiocManager,” “DOSE,” “clusterProfiler,” and “enrichplot” in the *R* language.

### 2.4. Molecular Docking

The 2D structures of key targets were downloaded from the PubChem database (https://pubchem.ncbi.nlm.nih.gov/). Chem3D was used to convert 2D structures into 3D structures and optimize the structures with minimum free energy. Active components that can interact with key targets were screened out through the network diagram of “drug-component-target,” and 3D structures of these active components were downloaded from the PDB database (https://www.rcsb.org/). Water molecules and small molecule ligands were removed in PyMOL. Autodock Tools was used for hydrogenation and active pocket location screening. Finally, Autodock Vina was employed for molecular docking to predict the binding sites and binding strength between the active components and key targets. The predicted binding sites were visualized by PyMOL, and the binding strength was evaluated by binding free energy. The lower binding free energy is, the higher binding strength will be.

### 2.5. Reagents

Antibodies of ATP-binding cassette transporter A1 (ABCA1) (ab66217), liver X receptor alpha (LXR*α*) (ab41902), and interleukin-1*β* (IL-1*β*) (ab234437) were purchased from Abcam (Cambridaeshire, UK). Antibodies of PPAR*γ* (16643-1-AP), ATP-binding cassette transporter G1 (ABCG1) (13578-1-AP), and platelet endothelial cell adhesion molecule-1 (CD31) (66065-2-Ig) were purchased from Proteintech Group (Wuhan, China). Antibodies of nuclear factor kappa-B p65 (NF-*κ*B p65) (8242), interleukin-6 (IL-6) (12912), and tumor necrosis factor alpha (TNF-*α*) (11948) were purchased from Cell Signaling Technology (Danvers, USA). Dulbecco's Modified Eagle's Medium (DMEM, 11965092), fetal bovine serum (FBS, 10100147), and penicillin-streptomycin (15140122) were obtained from Gibco (Thermo Fisher, Shanghai, China). Endothelial cell medium (ECM, 1001) was obtained from ScienCell (San Diego, USA). Human PPAR*γ* siRNA (si-PPAR*γ*, sc-29455) and mouse si-PPAR*γ* (sc-29456) were obtained from Santa Cruz Biotechnology (Santa Cruz, USA). Transfection reagent LipoRNAi (C0535) was bought from Beyotime Biotechnology (Shanghai, China). The human oxidized low-density lipoprotein (ox-LDL, YB-002) was acquired from Yiyuan Biotechnologies (Guangzhou, China), and human apolipoprotein A1 (ApoA-1) (178452) and HDL (LP3) were acquired from Merck Millipore (Burling, MA, USA). The pioglitazone (HY-13956) was obtained from MedChemExpress (Shanghai, China). The reverse transcription reagent (11141ES60) and amplification reagent (11202ES08) for real-time quantitative polymerase chain reaction (RT-qPCR) were purchased from Yeasen Biotechnology (Shanghai, China). The assay kit of cholesterol efflux (ab196985) was obtained from Abcam (Cambridaeshire, UK), and the assay kits of IL-1*β* (H002), IL-6 (H007-1-2), TNF-*α* (H052-1), total cholesterol (TC) (A111-1-1), low-density lipoprotein cholesterol (LDL-C) (A113-1-1), high-density lipoprotein cholesterol (HDL-C) (A112-2-1), and triglyceride (TG) (A110-1-1) were obtained from Jiancheng Bioengineering Institute (Nanjing, China).

### 2.6. Animals

40 male C57BL/6 (C57) mice and 30 male apolipoprotein e-deficient (ApoE^−/−^) mice were obtained from Model Animal Research Center of Nanjing University. All the mice were 6 to 8 weeks old and weighed about 20 grams. After 1 week of adaptive feeding, these mice were divided into 7 groups with 10 mice in each group: C57-ND group (C57 mice were fed with a normal diet combined with normal saline gavage), C57-HFD group (C57 mice were fed with a high-fat diet combined with normal saline gavage), C57-Low group (C57 mice were fed with a high-fat diet combined with low-dose QHZYF gavage), C57-High group (C57 mice were fed with a high-fat diet combined with high dose QHZYF gavage), ApoE-HFD group (ApoE^−/−^ mice were fed with a high-fat diet combined with normal saline gavage), ApoE-Low group (ApoE^−/−^ mice were fed with a high-fat diet combined with low-dose QHZYF gavage), and ApoE-High group (ApoE^−/−^ mice were fed with a high-fat diet combined with high dose QHZYF gavage). The high-fat diet contained 1.25% cholesterol and 40% fat (D12108C, Changzhou SYSE Bio-tech. Co., Ltd., Changzhou, China). The whole feeding process lasted for 12 weeks, with normal saline and QHZYF intragastric administration starting from the 9th week. QHZYF was diluted to 1 g/mL and 0.5 g/mL, the daily dose of the low-dose QHZYF group was 2.5 mg/g (0.5 g/mL QHZYF, 0.005 mL/g daily), and the daily dose of the high-dose QHZYF group was 5 mg/g (1 g/mL QHZYF, 0.005 mL/g daily). The whole experiment followed the US National Institutes of Health guidelines and was approved by the Animal Ethics Committee of Jiangsu Hospital of Chinese Medicine.

### 2.7. Histological Staining

The aortic root to aortic arch of mice were fixed with paraformaldehyde; HE, Masson, and Sirius red staining were performed by paraffin embedding; and the oil red O staining was performed by optimal cutting temperature (OCT) embedding. Then, HE, Masson, Sirius red, and oil red O staining were performed on the sections with corresponding staining solutions. The intact aortas were used to perform gross oil red staining, which were cut longitudinally after fixation with paraformaldehyde and then stained with an oil red staining solution.

### 2.8. Immunohistochemistry

The aortic root to aortic arch of mice were fixed with paraformaldehyde. Then, the following experimental steps were carried out in sequence: paraffin embedding, serial section, dewaxing, hydration, permeation, and antigen retrieval. The nonspecific binding site was blocked at room temperature with 5% bull serum albumin (BSA) for 1 hour. PPAR*γ*, LXR*α*, ABCA1, and ABCAG1 antibodies were diluted at the proportions recommended by the instructions and incubated overnight at 4°C. After that, they were incubated with the corresponding secondary antibody at room temperature for 1 hour. Finally, diaminobenzine (DAB) staining and nuclear staining were performed with DAB staining solution and hematoxylin, respectively.

### 2.9. Immunofluorescence

The basic steps were similar to immunohistochemistry. After blocking, the sections were incubated in the antibodies of NF-*κ*B p65, IL-1*β*, IL-6, TNF-*α*, and CD31 overnight at 4°C. Then, they were incubated in Alexa Fluor 488 or cyanine 3 (Cy3) coupled secondary antibodies at room temperature for 1 hour under dark condition. Finally, the cell nucleuses were stained with 4′,6-diamidino2-phenylindole (DAPI) staining solution, and antifade mounting medium was dropped onto the sections.

### 2.10. Cell Culture and Model Establishment

RAW 264.7 cells were provided by the Chinese Academy of Sciences (Shanghai, China). Human umbilical vein endothelial cells (HUVECs) were provided by Sigma-Aldrich (St. Louis, MO, USA). RAW 264.7 cells were cultured in DMEM supplemented with 10% FBS and 1% penicillin-treptomycin. HUVECs were cultured in ECM supplemented with 5% FBS and 1% penicillin-treptomycin. All cells were incubated in a 37°C incubator containing 5% carbon dioxide. The establishment of cell models was based on previously published literature and reagent supply manual, and RAW 264.7 cells were incubated with ox-LDL (80 *μ*g/mL) for 24 hours to establish the foam cell model. HUVECs were incubated with ox-LDL (80 *μ*g/mL) for 24 hours to establish the endothelial cell inflammation model. For the positive control in vitro experiments, RAW 264.7 cells and HUVECs were treated with pioglitazone (20 *μ*M) for 24 hours.

### 2.11. CCK-8 Cell Proliferation Assay

RAW 264.7 cells and HUVECs were cultured in 96-well plates, respectively. When the cell density in each well reached about 80%, the following different concentrations of QHZYF were, respectively, added to each well for 24 hours: 1 mg/mL, 2 mg/mL, 3 mg/mL, 4 mg/mL, 5 mg/mL, 6 mg/mL, 7 mg/mL, 8 mg/mL, 9 mg/mL, and 10 mg/mL. Then, 10 *μ*L CCK8 solution was added to each well. After 2 hours, the absorbance was measured at 450 nm.

### 2.12. Gene Knockdown

The siRNA transfection was performed when the cell density reached about 80%. LipoRNAi, si-PPAR*γ* (human or mouse), and serum-free medium were mixed according to the ratio recommended by the instructions. Then, the mixed solution was added into the corresponding cell culture dishes for 6 hours. After that, the serum-free medium was replaced with the complete medium and cultured for another 48 hours. The mRNA and protein expression of PPAR*γ* were detected by RT-qPCR and western blot.

### 2.13. Cell Oil Red O Staining

When RAW 264.7 cell density reached about 80%, they were fixed with paraformaldehyde for 10 minutes and then covered with staining wash buffer for 20 seconds. After that, the staining wash buffer was discarded, and oil red staining solution was added to soak cells for 30 minutes. Finally, the oil red staining solution was discarded, and the staining wash buffer was added to cover the cells for 30 seconds.

### 2.14. Cholesterol Efflux Assay

RAW 264.7 cells were cultured with fluorescent-labeled cholesterol reagent for 24 hours and then treated with ox-LDL or ox-LDL combined with QHZYF for 24 hours and serum-free medium containing ApoA-1 or HDL for another 4 hours. Finally, the fluorescence intensity of cell culture supernatant and intracellular lipid content was determined by multifunctional microplate reader (Ex/Em = 485/523 nm). The cholesterol efflux rate was equal to the fluorescence intensity of cell culture supernatant divided by the sum of the fluorescence intensity of cell culture supernatant and intracellular lipid content.

### 2.15. Western Blot

Protein extraction reagents were used to extract proteins from cells or mouse aortas, and electrophoresis was performed using SDS-polyacrylamide gel to isolate proteins. Then, the protein was transferred to polyvinylidene fluoride (PVDF) membrane, blocked with 5% defatted milk powder for 1 hour, and incubated with PPAR*γ*, LXR*α*, ABCA1, ABCG1, and NF-*κ*B p65 primary antibodies at 4°C overnight. The corresponding rabbit or mouse secondary antibodies were incubated at room temperature for 1 hour. The chemiluminescence reaction was performed using the ECL kit, and protein blot was detected by the Bio-Rad gel imaging system.

### 2.16. RT-qPCR

Total RNA was extracted by TRIzol method, and the concentration of total RNA was determined. Hifair III 1st Strand cDNA Synthesis SuperMix was used for reverse transcription of 1 *μ*g total RNA, and the reverse transcription program was set as follows: 25°C for 5 minutes, 55°C for 15 minutes, and 85°C for 5 minutes. Hieff SYBR Green Master Mix was used for PCR amplification, and the amplification procedure was set as follows: predenaturation for 5 minutes at 95°C, followed by 40 cycles of 95°C for 10 seconds and 60°C for 30 seconds. The target mRNA expression level was calculated by the △△Ct method with GAPDH as internal reference. The primer sequences are shown in Supplementary Table S2. Primer synthesis was completed by Generay Biotech (Shanghai, China).

### 2.17. ELISA and Serum Lipid Detection

The concentrations of IL-1*β*, IL-6, and TNF-*α* in serum or cell culture supernatant were determined by the competitive method. Samples and standard substances with different concentration gradients were added to the enzyme-labeled wells precoated with antibodies, followed by biotin-labeled recognition antigen, avidin-HRP, chromogenic solution, and termination solution. Absorbance was measured at 450 nm, and sample concentration was calculated according to the fitting curve of standard substances. The concentrations of serum TC, LDL-C, HDL-C, and TG were determined using corresponding assay kits. Samples and standard are added to the 96-well plate, and then the working liquid was added for reaction. Finally, the absorbance was measured at the corresponding wavelength, and the sample concentration was calculated according to the formula provided in the instruction.

### 2.18. Statistical Analysis

All data were statistically analyzed by GraphPad Prism 7.0 and expressed as mean ± standard deviation (SD). One-way ANOVA was performed to compare three or more groups of data. *P* value less than 0.05 (*P* < 0.05) was considered statistically significant.

## 3. Results

### 3.1. UPLC-Q-TOF/MS Combined with Network Pharmacology and Bioinformatics Analysis Predicted the Potential Targets of QHZYF in the Treatment of Atherosclerosis

**Figure 1:**
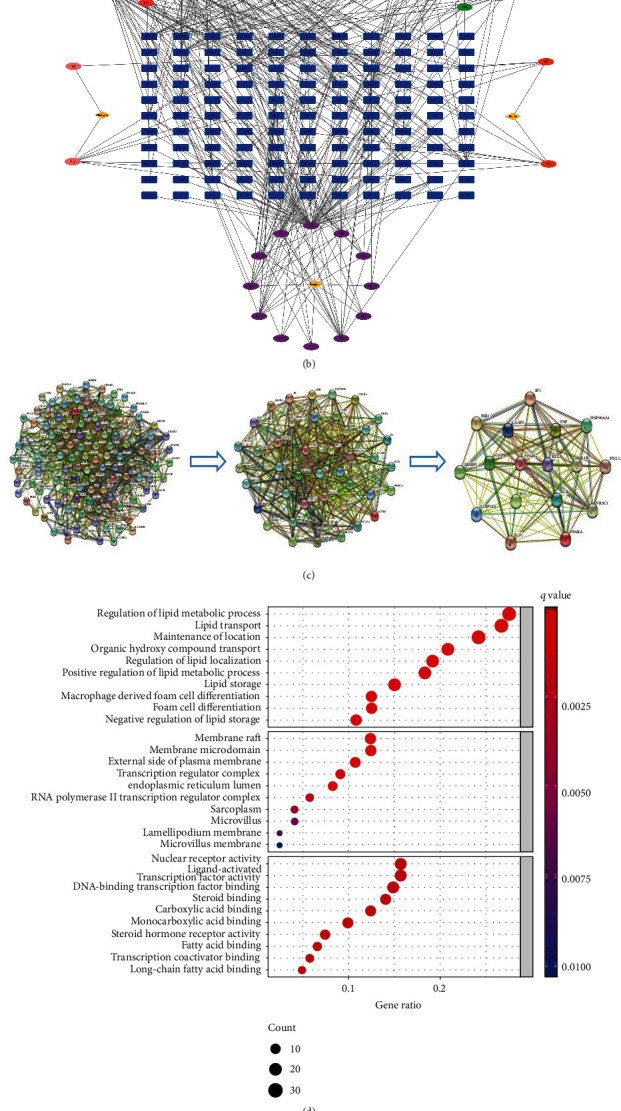
Identification of chemical components and prediction of targets of QHZYF. (a) The chromatograms of QHZYF in positive and negative ion modes (red: positive ion mode, green: negative ion mode). (b) The network diagram of “drug-component-target” (blue rectangular boxes represent the targets, yellow diamond boxes show the five kinds of Chinese herbs contained in QHZYF, and circular boxes around the yellow diamond boxes indicate the chemical components contained in each Chinese herb). (c) The PPI network diagram (the network core targets were obtained by two network centrality analyses). (d) The bubble chart of GO analysis (the top 30 biological processes were shown). (e) The bubble chart of KEGG analysis (the top 30 pathways were shown).

In order to predict the potential targets of QHZYF in the treatment of atherosclerosis, we first used UPLC-Q-TOF/MS technology to identify the active components of QHZYF in positive and negative ion modes. A total of 78 active ingredients were found, including Genipin gentiobioside, D-mannoheptulose, and 10-O-Methyl protosappanin b (Figure 1(a) and Supplementary Table. S1). Then, we predicted the potential targets of these active ingredients in TCMSP and BATMAN databases and intersected them with atherosclerotic therapeutic targets screened from DrugBank, GeneCards, OMIM, TTD, and PharmGKB databases. The “drug-component-target” network diagram was obtained, and the blue rectangular boxes in the diagram were the potential targets of QHZYF in the treatment of atherosclerosis (Figure 1(b)). In order to further identify the core targets, we used the STRING database to construct PPI network diagram of potential targets (Figure 1(c)) and conducted GO and KEGG enrichment analyses using bioinformatics analysis technology (Figures 1(d) and 1(e)). These results showed that the core targets of QHZYF in the treatment of atherosclerosis include PPARG (gene name of PPAR*γ*), RELA (gene name of NF-*κ*B p65), and IL-1B (gene name of IL-1*β*), the core pathways include PPARG and NF-*κ*B signaling pathways, and the core biological processes include lipid metabolism and transport. PPAR*γ* is widely distributed in macrophages, smooth muscle cells, and endothelial cells, playing an antiatherosclerosis role in many aspects. PPAR*γ* can affect the cholesterol efflux function through regulating downstream LXR*α*, ABCA1, and ABCG1 molecules and can also affect the expression of inflammatory factor IL-1*β*, IL-6, and TNF-*α* in endothelial cells through regulating downstream NF-*κ*B p65 molecule [13, 14]. Therefore, we speculated that PPAR*γ* is the core target of QHZYF in the treatment of atherosclerosis. QHZYF may regulate the PPAR*γ*/LXR*α*/ABCA1-ABCG1 pathway and PPAR*γ*/NF-*κ*B p65 pathway by targeting PPAR*γ*, thus affecting cholesterol efflux and endothelial cell inflammation to alleviate atherosclerosis.

### 3.2. Molecular Docking Simulated the Binding Sites and Binding Strength of Active Components in QHZYF to PPAR*γ*

**Figure 2:**
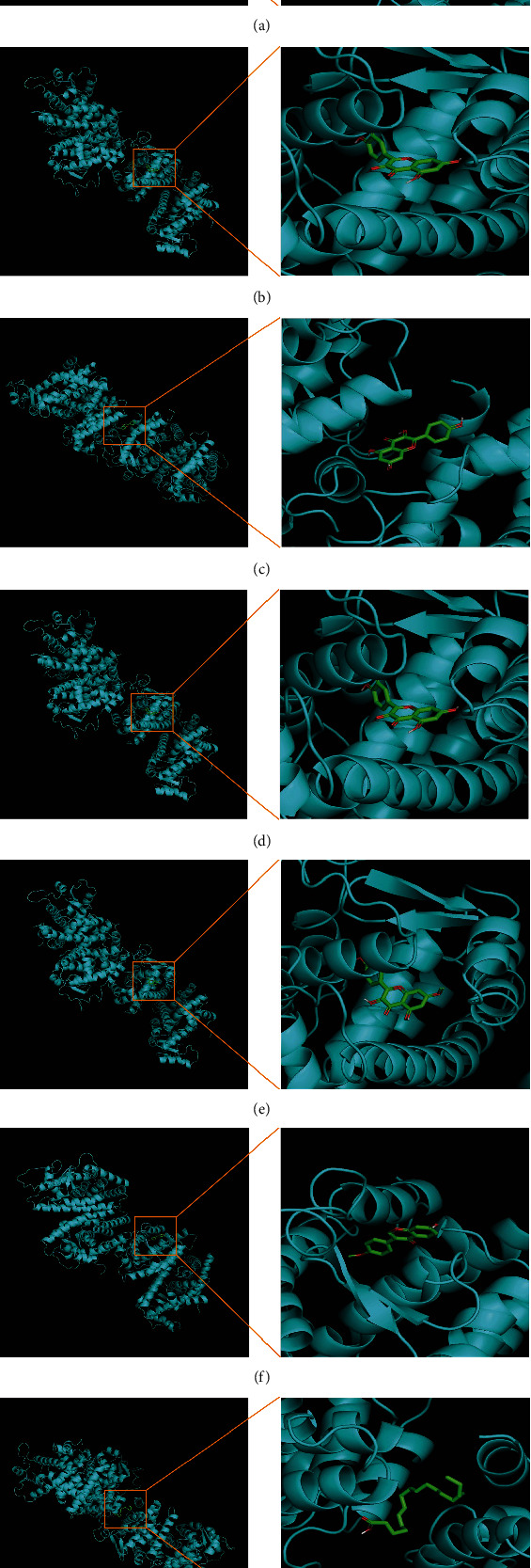
Prediction of binding sites and binding modes between PPAR*γ* and chemical components contained in QHZYF. (a) Prediction of binding site and binding mode between PPAR*γ* and sucrose. (b) Prediction of binding site and binding mode between PPAR*γ* and calycosin. (c) Prediction of binding site and binding mode between PPAR*γ* and kaempferol. (d) Prediction of binding site and binding mode between PPAR*γ* and isorhamnetin. (e) Prediction of binding site and binding mode between PPAR*γ* and rhamnocitrin. (f) Prediction of binding site and binding mode between PPAR*γ* and formononetin. (g) Prediction of binding site and binding mode between PPAR*γ* and linolenic acid.

In order to verify whether the active ingredients in QHZYF can directly act on PPAR*γ*, we found 7 components that may have an interactive relationship with PPAR*γ* from the “drug-component-target” network diagram (i.e., sucrose, calycosin, kaempferol, isorhamnetin, rhamnocitrin, formononetin, and linolenic acid). Next, we used molecular docking technology to simulate the binding of these active components to PPAR*γ*. The results showed that all of these components had potential binding sites with PPAR*γ* (Figures 2(a)–2(g)). Their binding free energy was listed as follows in ascending order: formononetin (-8.3), isorhamnetin (-8.2), rhamnocitrin (-8.2), kaempferol (-8.0), calycosin (-7.7), sucrose (-5.9), and linolenic acid (-5.4). Subsequently, we conducted a series of in vivo and in vitro experiments to examine the efficacy and mechanism of QHZYF in treating atherosclerosis.

### 3.3. QHZYF Attenuates Atherosclerosis In Vivo

**Figure 3:**
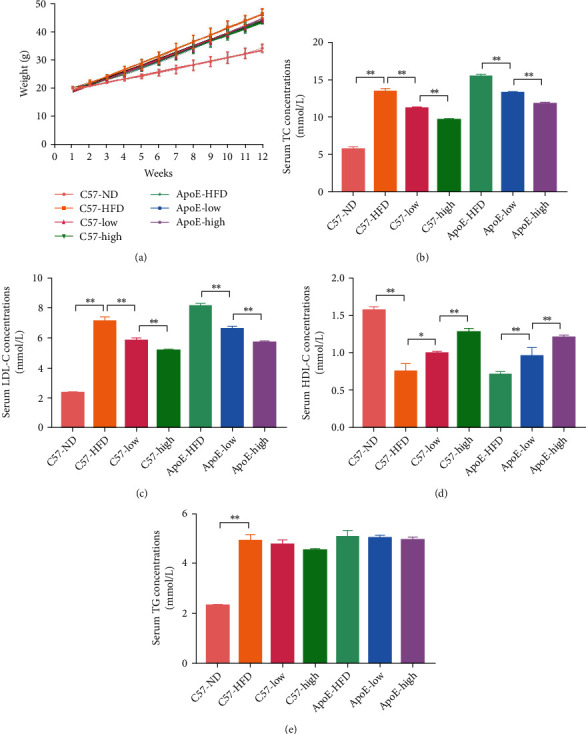
The effects of QHZYF on body weight and concentrations of serum lipid in ApoE^−/−^ mice and C57 wild-type mice. (a) The trends of body weight from week 1 to week 12 in mice. (b) The effects of QHZYF on concentrations of TC in serum. (c)The effects of QHZYF on concentrations of LDL-C in serum. (d) The effects of QHZYF on concentrations of HDL-C in serum. (e) The effects of QHZYF on concentrations of TG in serum. (^∗^*P* < 0.05, ^∗∗^*P* < 0.01).

**Figure 4:**
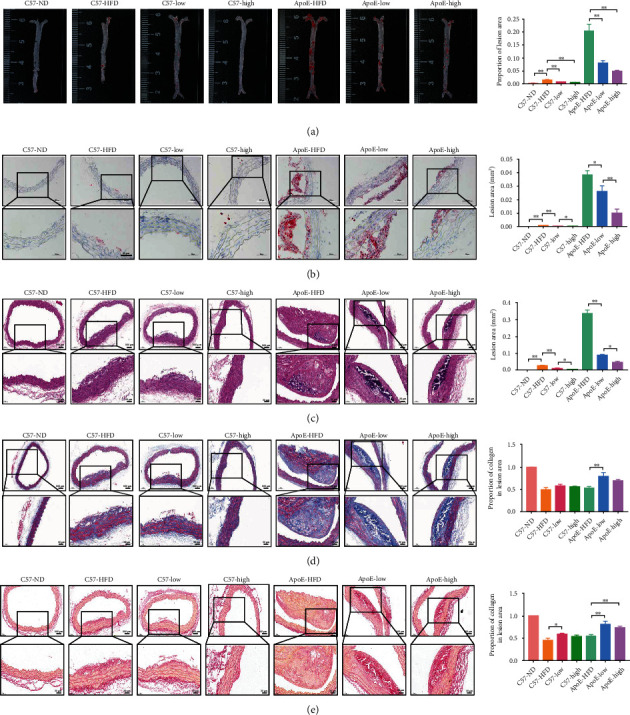
The effects of QHZYF on atherosclerosis in ApoE^−/−^ mice and C57 wild-type mice. (a) Representative images of gross oil red staining of aortas and quantitative analysis of the percentage of lesions area in entire aorta (*n* = 3). (b) Representative images of oil red O staining of aortic root and quantitative analysis of the percentage of lesions area in aortic root section area (*n* = 3). (c) Representative images of HE staining of aortic root and quantitative analysis of the percentage of lesions area in aortic root section area (*n* = 3). (d) Representative images of Masson staining of aortic root and quantitative analysis of the percentage of collagen fiber area in plaque (*n* = 3). (e) Representative images of Sirius red staining of aortic root and quantitative analysis of the percentage of collagen fiber area in plaque (*n* = 3) (^∗^*P* < 0.05, ^∗∗^*P* < 0.01).

We first studied the effect of QHZYF in the treatment of atherosclerosis. In order to exclude the possible interference of apolipoprotein e-deficient on the efficacy, both C57 mice and ApoE^−/−^ mice were used in this study. After 12 weeks of feeding, we found that QHZYF had no significant effect on the body weight of mice (Figure 3(a)). Lipid detection results of mice serum showed that QHZYF could significantly reduce the concentrations of LDL-C and TC and increase the concentrations of HDL-C in a dose-dependent manner but had no significant effect on TG concentrations (Figures 3(b)–3(e)). The results of gross oil red staining suggested that, compared with the high-fat diet groups, both low-dose and high-dose QHZYF could reduce the plaque load in the aortas of ApoE^−/−^ mice (Figure 4(a)). However, there was no significant difference in the effect of low-dose and high-dose QHZYF. The following results of oil red O staining and HE staining of the aortic root manifested that QHZYF could significantly reduce the plaque area in the aortic vascular lumen of ApoE^−/−^ mice, and its efficacy was dose-dependent (Figures 4(b) and 4(c)). Interestingly, we observed some small atherosclerotic plaques in high-fat fed C57 mice and found that the plaque area gradually decreased as the dose of QHZYF increased. However, C57 mice are known to have difficulty developing atherosclerotic plaques even when fed with a high-fat diet. Since the plaques were small, we could not rule out the possibility that they were nonspecific stains. Therefore, the results of the C57 mice should be interpreted with caution. Finally, we evaluated the effect of QHZYF on collagen fiber content in plaques by Masson staining and Sirius red staining. The results indicated that QHZYF could increase the content of collagen fiber in ApoE^−/−^ mice, but the effects on C57 mice were not clear (Figures 4(d) and 4(e)). In summary, the above results suggest that QHZYF can delay the progression of atherosclerosis.

### 3.4. QHZYF Regulates Cholesterol Efflux through PPAR*γ*/LXR*α*/ABCA1-ABCG1 Pathway In Vivo

**Figure 5:**
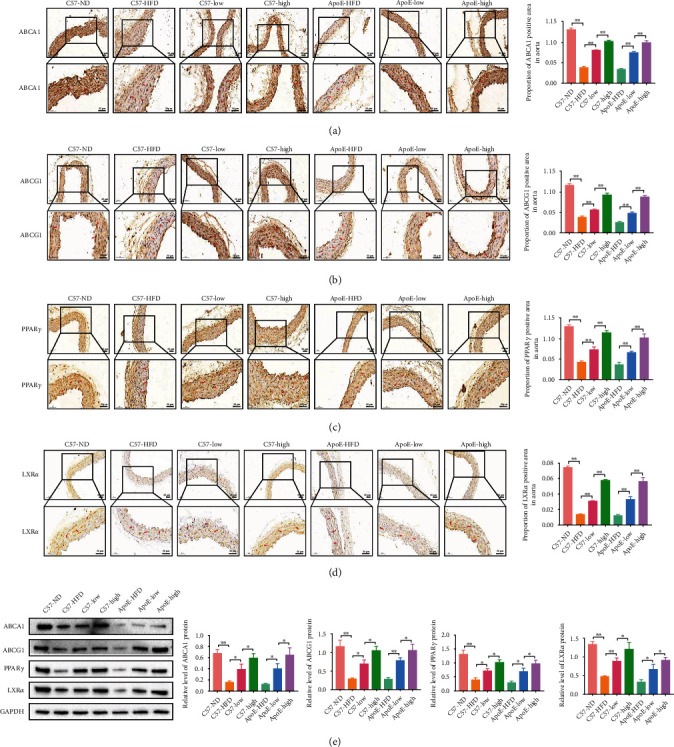
The effects of QHZYF on the expression levels of ABCA1, ABCG1, PPAR*γ*, and LXR*α* in aorta. (a–d) Immunohistochemical representative images of the expression levels of ABCA1, ABCG1, PPAR*γ*, and LXR*α* in aorta and quantitative analysis of the percentage of positive area in aorta (*n* = 3). (e) The expression levels of ABCA1, ABCG1, PPAR*γ*, and LXR*α* in aorta were detected by western blot and quantitative analysis of the gray value (*n* = 3) (^∗^*P* < 0.05, ^∗∗^*P* < 0.01).

Next, we studied the potential mechanism of QHZYF in the treatment of atherosclerosis in vivo. Firstly, we detected the expression levels of related proteins in the PPAR*γ*/LXR*α*/ABCA1-ABCG1 pathway in aortas by immunohistochemical method. The results showed that the expression levels of PPAR*γ*, LXR*α*, ABCA1, and ABCG1 in aortas of both C57 and ApoE^−/−^ mice were significantly downregulated after 12 weeks of high-fat diet, while QHZYF could significantly upregulate the levels of these proteins in a dose-dependent manner (Figures 5(a)–5(d)). After that, we further verified these results by western blot, and similar findings were obtained. The expression levels of PPAR*γ*, LXR*α*, ABCA1, and ABCG1 in the aortas of the high-fat diet groups were significantly decreased, while QHZYF could reverse these trends (Figure 5(e)). These results suggested that the mechanism of QHZYF alleviating atherosclerosis may be related to activating the PPAR*γ*/LXR*α*/ABCA1-ABCG1 pathway to regulate cholesterol efflux.

### 3.5. QHZYF Inhibits Endothelial Cell Inflammation through PPAR*γ*/NF-*κ*B p65 Pathway In Vivo

**Figure 6:**
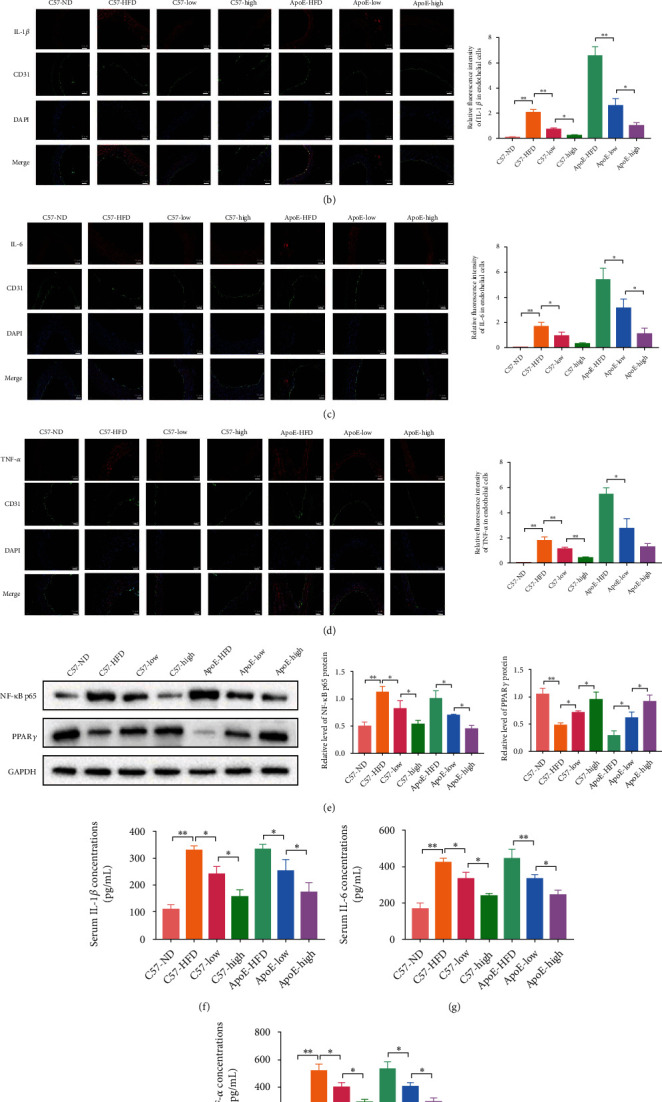
The effects of QHZYF on the expression levels of NF-*κ*B p65 in aortic endothelial cells and the concentrations of IL-1*β*, IL-6, and TNF-*α* in serum (red: NF-*κ*B p65, IL-1*β*, IL-6 or TNF-*α*, green: CD31, blue: DAPI). (a–d) Representative images of immunofluorescence of the expression levels of NF-*κ*B p65, IL-1*β*, IL-6, and TNF-*α* in aortic endothelial cell and quantitative analysis of the intensity of red fluorescence in endothelial cells (*n* = 3). (e) The expression levels of NF-*κ*B p65 and PPAR*γ* in aorta were detected by western blot and quantitative analysis of the gray value (*n* = 3). (f–h) The concentrations of IL-1*β*, IL-6, and TNF-*α* in serum were detected by ELISA (^∗^*P* < 0.05, ^∗∗^*P* < 0.01).

In order to study the effect of QHZYF on endothelial cell inflammation mediated by PPAR*γ*/NF-*κ*B p65 pathway, we conducted fluorescence colocalization of endothelial cell markers CD31, NF-*κ*B p65, IL-1*β*, IL-6, and TNF-*α* through immunofluorescence technique. The results showed that the expressions levels of NF-*κ*B p65, IL-1*β*, IL-6, and TNF-*α* colocated with CD31 were significantly upregulated in both C57 and ApoE^−/−^ mice after 12 weeks of high-fat diet, suggesting that high-fat diet induced NF-*κ*B p65-related inflammatory responses in aortic endothelial cells. In the QHZYF groups, the expression levels of these inflammatory factors were significantly downregulated in a dose-dependent manner (Figures 6(a)–6(d)). Later, to further verify these results, the expression levels of NF-*κ*B p65 and PPAR*γ* in the aorta were determined by western blot, and the concentrations of IL-1*β*, IL-6, and TNF-*α* in serum were determined by ELISA. The results were consistent with those of immunofluorescence; that is, high-fat diet led to the increase of these inflammatory factors, and QHZYF could significantly reverse these trends (Figures 6(e)–6(h)). These results manifested that another possible mechanism of QHZYF treating atherosclerosis is the inhibition of endothelial cell inflammation by targeting the PPAR*γ*/NF-*κ*B p65 pathway.

### 3.6. QHZYF Regulates Cholesterol Efflux through PPAR*γ*/LXR*α*/ABCA1-ABCG1 Pathway In Vitro

#### 3.6.1. QHZYF Reduced the Intracellular Lipid Content, Increased Cholesterol Efflux Rate, and Upregulated the Expression Levels of Related Proteins and MRNA in PPAR*Γ*/LXR*Α*/ABCA1-ABCG1 Pathway in RAW 264.7 Cells

**Figure 7:**
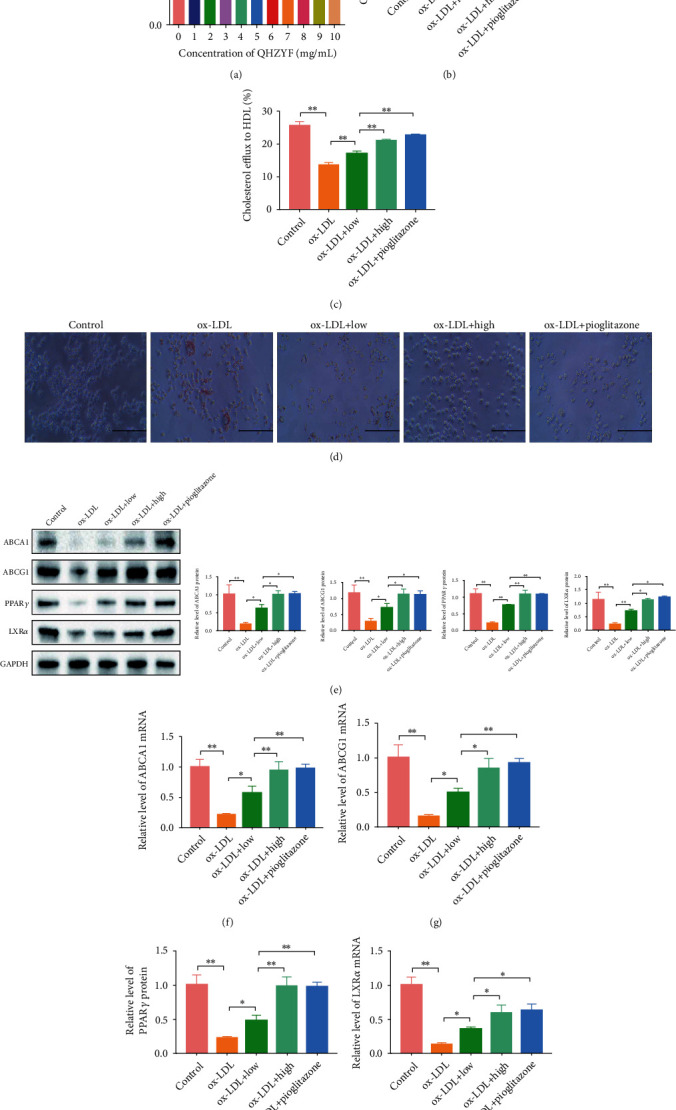
The effects of QHZYF on lipid content, cholesterol efflux rate, and expression levels of ABCA1, ABCG1, PPAR*γ*, and LXR*α* in RAW 264.7 cells. (a) The effect of QHZYF on RAW 264.7 cell viability. (b, c) The effects of QHZYF on cholesterol efflux to ApoA-1 and HDL. (d) Representative images of oil red O staining for RAW 264.7 cells. (e) The protein expression levels of ABCA1, ABCG1, PPAR*γ*, and LXR*α* in RAW 264.7 cells were detected by western blot and quantitative analysis of the gray value (*n* = 3). (f–i) The mRNA expression levels of ABCA1, ABCG1, PPAR*γ*, and LXR*α* in RAW 264.7 cells were detected by RT-qPCR (^∗^*P* < 0.05, ^∗∗^*P* < 0.01).

To further verify whether QHZYF affects the progression of atherosclerosis through the above mechanisms, we carried out in vitro experiments. Conversion of RAW 264.7 cells into foam cells induced by ox-LDL is a common cell model to study the mechanism of cholesterol efflux in vitro. First, we screened the suitable concentration range of QHZYF for RAW 264.7 cells through CCK-8 cell proliferation assay. The results indicated that QHZYF had inhibitory effect on RAW 264.7 cells when the concentration was more than 8 mg/mL (Figure 7(a)). Therefore, 7 mg/mL and 3.5 mg/mL were selected as the high and low doses in our subsequent experiments. Afterwards, ox-LDL or ox-LDL combined with QHZYF was used to intervene RAW 264.7 cells to study the effect of QHZYF on atherosclerotic foam cells in vitro. Meanwhile, RAW 264.7 cells treated with pioglitazone (a selective PPAR*γ* agonist) were used as the positive control group. The results of cholesterol efflux assay manifested that ox-LDL reduced the cholesterol efflux to ApoA-1 and HDL, while QHZYF and pioglitazone could increase the cholesterol efflux, and the effect of high dose of QHZYF was similar to that of pioglitazone (Figures 7(b) and 7(c)). Next, the results of cell oil red O staining showed that ox-LDL significantly increased the lipid content in RAW 264.7 cells, while QHZYF and pioglitazone could reduce the lipid content in cells (Figure 7(d)). Then, we measured the expression levels of PPAR*γ*, LXR*α*, ABCA1, and ABCG1 in cells by western blot, and the results showed that ox-LDL significantly downregulated the expression levels of these proteins, while QHZYF and pioglitazone could reverse these trends, and the high dose of QHZYF played similar role with that of pioglitazone (Figure 7(e)). Finally, we further verified these results from mRNA level by RT-qPCR. The results were consistent with those of western blot, ox-LDL significantly reduced the mRNA expression of PPAR*γ*, LXR*α*, ABCA1 and ABCG1 in cells, while QHZYF and pioglitazone could increase the mRNA expression levels (Figures 7(f)–7(i)).

#### 3.6.2. PPAR*γ* Knockdown Reversed the Effects of QHZYF on Intracellular Lipid Content, Cholesterol Efflux Rate, and PPAR*γ*/LXR*α*/ABCA1-ABCG1 Pathway

**Figure 8:**
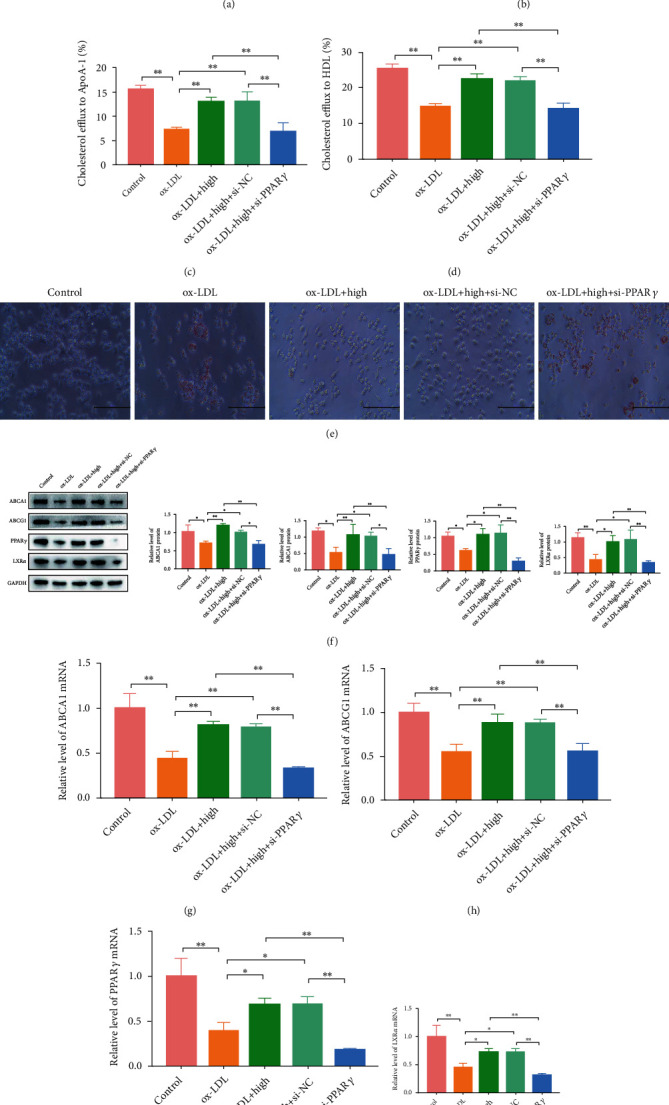
PPAR*γ* knockdown reversed the effects of QHZYF on lipid content, cholesterol efflux rate, and expression levels of ABCA1, ABCG1, PPAR*γ*, and LXR*α* in RAW 264.7 cells. (a) The effect of PPAR*γ* knockdown on the protein expression level was detected by western blot and quantitative analysis of the gray value (*n* = 3). (b) The effect of PPAR*γ* knockdown on the mRNA expression level was detected by RT-qPCR. (c, d) The effects of QHZYF on cholesterol efflux to ApoA-1 and HDL after PPAR*γ* knockdown. (e) Representative images of oil red O staining for RAW 264.7 cells. (f) The protein expression levels of ABCA1, ABCG1, PPAR*γ*, and LXR*α* in RAW 264.7 cells were detected by western blot and quantitative analysis of the gray value (*n* = 3). (g–j) The mRNA expression levels of ABCA1, ABCG1, PPAR*γ*, and LXR*α* in RAW 264.7 cells were detected by RT-qPCR (^∗^*P* < 0.05, ^∗∗^*P* < 0.01).

In order to further investigate whether the mechanism of QHZYF in reducing lipid content and increasing cholesterol efflux is related to PPAR*γ*/LXR*α*/ABCA1-ABCG1 pathway, we constructed PPAR*γ* knockdown RAW 264.7 cell model in vitro to observe whether the effects of QHZYF on intracellular lipid content, cholesterol efflux rate, and expression levels of downstream proteins are reversed. 48 hours after si-PPAR*γ* was transfected into RAW 264.7 cells, we found that the expression levels of protein and mRNA of PPAR*γ* were indeed significantly inhibited via western blot and RT-qPCR, while cells transfected with si-NC showed no significant changes (Figures 8(a) and 8(b)). After that, the normal or PPAR*γ* knockdown cells were treated with ox-LDL or ox-LDL combined with QHZYF. The detection results of cholesterol efflux found that the effects of QHZYF on cholesterol efflux were significantly weakened after PPAR*γ* gene was knocked down (Figures 8(c) and 8(d)). The results of oil red O staining showed that ox-LDL increased the lipid content in normal cells, while QHZYF could reverse the effects of ox-LDL. However, the effects of QHZYF were significantly weakened in PPAR*γ* knockdown cells (Figure 8(e)). The western blot results manifested that the upregulation effects of QHZYF on PPAR*γ*, LXR*α*, ABCA1, and ABCG1 protein were significantly diminished after PPAR*γ* knockdown (Figure 8(f)). Consistent results were obtained in subsequent RT-qPCR results; after PPAR*γ* knockdown, the effects of QHZYF on related mRNA were weakened significantly (Figures 8(g)–8(j)). All the above results suggest that QHZYF could reduce the lipid content and increase cholesterol efflux rate in vitro, and the mechanism depends on the PPAR*γ*/LXR*α*/ABCA1-ABCG1 pathway.

### 3.7. QHZYF Inhibits Endothelial Cell Inflammation through PPAR*γ*/NF-*κ*B p65 Pathway In Vitro

#### 3.7.1. QHZYF Upregulated the Expression Levels of PPAR*γ* and Downregulated the Expression Levels of NF-*κ*B p65, IL-1*β*, IL-6, and TNF-*α* in HUVECs

**Figure 9:**
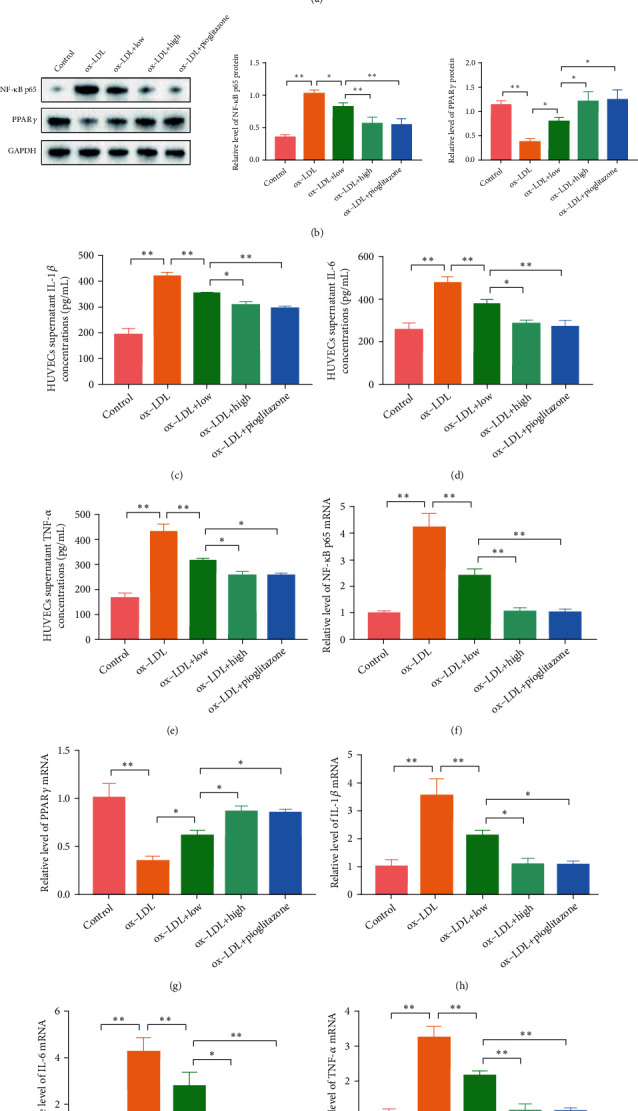
The effects of QHZYF on the expression levels of NF-*κ*B p65, PPAR*γ*, IL-1*β*, IL-6, and TNF-*α* in HUVECs. (a) The effect of QHZYF on HUVEC viability. (b) The protein expression levels of NF-*κ*B p65 and PPAR*γ* in HUVECs were detected by western blot and quantitative analysis of the gray value (*n* = 3). (c–e) The concentrations of IL-1*β*, IL-6, and TNF-*α* in HUVEC culture supernatant were detected by ELISA. (f–j) The mRNA expression levels of NF-*κ*B p65, PPAR*γ*, IL-1*β*, IL-6, and TNF-*α* in HUVECs were detected by RT-qPCR (^∗^*P* < 0.05, ^∗∗^*P* < 0.01).

Next, we studied the efficacy and mechanism of QHZYF in improving endothelial cell inflammation in vitro. Ox-LDL stimulation of HUVECs is a common cell model to study endothelial cell inflammation in vitro. Following the practice in previous studies, we firstly screened the suitable concentration range of QHZYF for HUVECs through CCK8 experiment. The results showed that QHZYF had an inhibitory effect on HUVECs when it was more than 5 mg/mL (Figure 9(a)). Therefore, 4 mg/mL and 2 mg/mL were selected as the high and low doses in our subsequent studies. Whereafter, ox-LDL or ox-LDL combined with QHZYF was used to treat HUVECs to study the effect of QHZYF on endothelial cell inflammation. Meanwhile, HUVECs treated with pioglitazone were used as the positive control group. The results of western blot showed that ox-LDL significantly downregulate the level of PPAR*γ* and upregulate the level of NF-*κ*B p65, while QHZYF and pioglitazone could reverse these trends, and the effect of high dose of QHZYF was similar to that of pioglitazone (Figure 9(b)). Then, we assayed the concentrations of IL-1*β*, IL-6, and TNF-*α* in HUVEC culture supernatant by ELISA, and the results indicated that ox-LDL significantly increased the concentrations of IL-1*β*, IL-6, and TNF-*α*, while QHZYF and pioglitazone could reduce the levels of these inflammatory factors, and the high dose of QHZYF played a similar role with that of pioglitazone (Figures 9(c)–9(e)). Finally, we further verified these results from the mRNA level through RT-qPCR. The results were coherent with those of western blot and ELISA and ox-LDL significantly reduced the mRNA level of PPAR*γ* in HUVECs and increased the mRNA level of NF-*κ*B p65, IL-1*β*, IL-6, and TNF-*α*, while QHZYF and pioglitazone could increase the mRNA level of PPAR*γ* and reduce mRNA levels of NF-*κ*B p65, IL-1*β*, IL-6, and TNF-*α* (Figures 9(f)–9(j)).

#### 3.7.2. PPAR*γ* Knockdown Reversed the Effects of QHZYF on PPAR*γ*/NF-*κ*B p65 Pathway and Downstream Inflammatory Factors IL-1*β*, IL-6, and TNF-*α*

**Figure 10:**
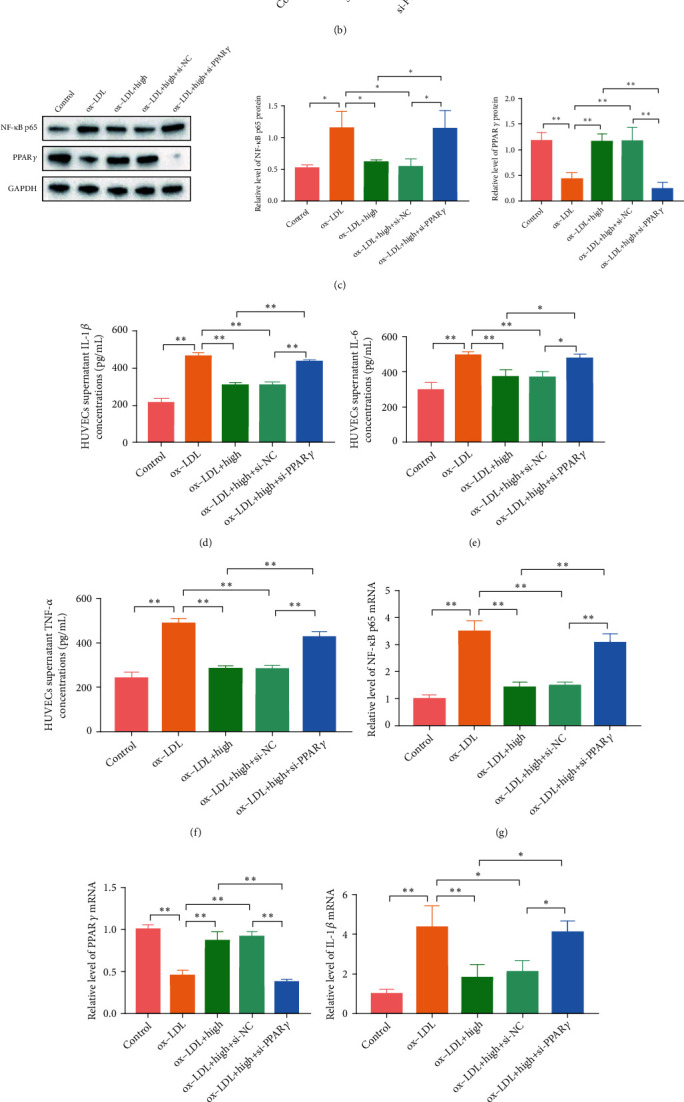
PPAR*γ* knockdown reversed the effects of QHZYF on the expression levels of NF-*κ*B p65, PPAR*γ*, IL-1*β*, IL-6, and TNF-*α* in HUVECs. (a) The effect of PPAR*γ* knockdown on the protein expression level was detected by western blot and quantitative analysis of the gray value (*n* = 3). (b) The effect of PPAR*γ* knockdown on the mRNA expression level was detected by RT-qPCR. (c) The protein expression levels of NF-*κ*B p65 and PPAR*γ* in HUVECs were detected by western blot and quantitative analysis of the gray value (*n* = 3). (d–f) The concentrations of IL-1*β*, IL-6, and TNF-*α* in HUVEC culture supernatant were detected by ELISA. (g–k) The mRNA expression levels of NF-*κ*B p65, PPAR*γ*, IL-1*β*, IL-6, and TNF-*α* in HUVECs were detected by RT-qPCR (^∗^*P* < 0.05, ^∗∗^*P* < 0.01).

In order to further verify whether the mechanism of QHZYF in inhibiting endothelial cell inflammation is related to PPAR*γ*/NF-*κ*B p65 pathway, we constructed the PPAR*γ* knockdown HUVEC model in vitro to observe whether the effects of QHZYF on NF-*κ*B p65 and downstream inflammatory factors are reversed. 48 hours after si-PPAR*γ* was transfected into HUVECs, we found that the expression levels of protein and mRNA of PPAR*γ* were indeed significantly inhibited by western blot and RT-qPCR, while cells transfected with si-NC showed no significant changes (Figures 10(a) and 10(b)). Then, the normal or PPAR*γ* knockdown cells were treated with ox-LDL or ox-LDL combined with QHZYF. The western blot results indicated that the upregulation effects of QHZYF on PPAR*γ* and the downregulation effects on NF-*κ*B p65 protein were significantly diminished after PPAR*γ* knockdown (Figure 10(c)). The results of ELISA showed that ox-LDL increased the concentrations of IL-1*β*, IL-6, and TNF-*α* in normal cells, while QHZYF could reduce the concentrations of these inflammatory factors in a dose-dependent manner. However, the effects of QHZYF were significantly weakened in PPAR*γ* knockdown cells (Figures 10(d)–10(f)). Consistent results were obtained in subsequent RT-qPCR experiments; after PPAR*γ* gene was knockdown, the effects of QHZYF on related mRNA were significantly weaken (Figures 10(g)–10(k)). These results suggested that QHZYF could inhibit HUVEC inflammation in vitro, and the mechanism depends on the PPAR*γ*/NF-*κ*B p65 pathway.

**Figure 11:**
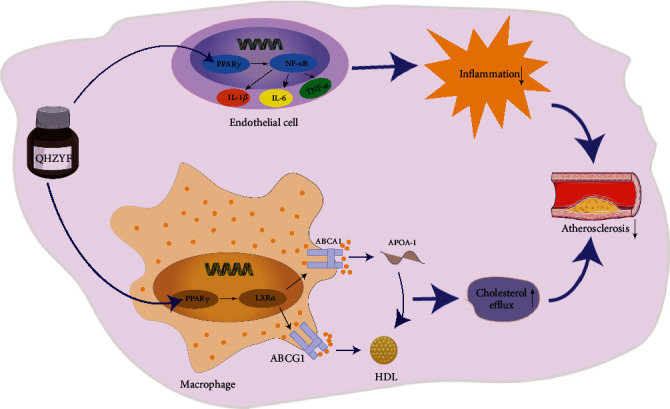
The mechanism of QHZYF in treating atherosclerosis. Through targeted regulation of PPAR*γ* in macrophages and endothelial cells, QHZYF activates PPAR*γ*-mediated PPAR*γ*/LXR*α*/ABCA1-ABCG1 and PPAR*γ*/NF-*κ*B pathways, resulting in increased cholesterol efflux in macrophages and decreased inflammatory response in endothelial cells, thus ultimately attenuating atherosclerosis.

**Figure 12:**
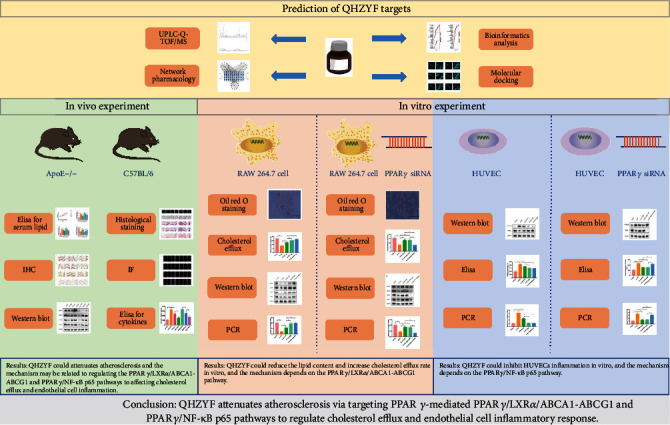
Graphic abstract of this study.

Based on the above results, this study confirmed that QHZYF could attenuate atherosclerosis via targeting PPAR*γ*-mediated PPAR*γ*/LXR*α*/ABCA1-ABCG1 and PPAR*γ*/NF-*κ*B p65 pathways to regulate cholesterol efflux and endothelial cell inflammatory response. The pharmacological mechanisms found in this study, and the overall research process is summarized in Figures 11 and 12.

## 4. Discussion

Nowadays, new drugs continue to emerge, providing more choices for the treatment of atherosclerosis, but the prevention and treatment of atherosclerosis are still facing a grim situation. The main reason is that current drugs mainly affect atherosclerosis from three aspects including cholesterol synthesis, absorption, and degradation, while the intervention effect on other pathogenesis of atherosclerosis is limited. It is a heated and difficult point to find out the way to solve the residual problems beyond existing drug therapy. Recently, there has been increasing evidence that cholesterol efflux dysfunction and endothelial inflammation are among the major residual risks in addition to current cholesterol-lowering drug therapy [15, 16]. Improving cholesterol efflux function and inhibiting endothelial inflammation are expected to be important strategies for the treatment of atherosclerosis in the future.

The process of cholesterol efflux is mainly regulated by ABCA1 and ABCG1, which transport cholesterol to ApoA-1 and HDL, respectively [17]. Studies have shown that the decreased activity of ABCA1 and ABCG1 proteins can lead to a large amount of cholesterol deposition in cells and increase the risk of atherosclerosis, while the increased activity of these two proteins can enhance the efficiency of cholesterol efflux and reduce the risk of atherosclerosis [18]. Targeting ABCA1 or ABCG1 is a promising therapeutic direction. However, there is still a lack of drugs that can directly activate ABCA1 or ABCG1; meanwhile, the treatment via inhibiting ABCA1 or ABCG1 protein degradation has only shown preliminary effects, and further studies are needed [19, 20]. Another approach to regulate the cholesterol efflux process is to target the upstream molecules of ABCA1 and ABCG1. LXR*α* is a transcription regulator of ABCA1 and ABCG1. LXR*α* can form heterodimer with retinoid X receptor (RXR) and then bind to LXR reflection elements in the gene promoter region to induce the production of ABCA1 and ABCG1 [21]. However, clinical and animal trials of LXR*α* agonists in the treatment of atherosclerosis have not achieved the desired effect. Some adverse effects, including central neurotoxicity, elevated levels of very low-density lipoprotein (VLDL), and triglycerides, are the main reasons that hinder its application [22–24]. Therefore, more intervention targets related to cholesterol efflux remain to be further explored. In terms of endothelial inflammation, although endothelial dysfunction caused by endothelial inflammation is the initial stage of atherosclerosis and plays an important role in the progression, most of the previous studies have focused on systemic inflammation, while few studies were mainly concerned with endothelial inflammation, and effective drugs are still lacking at present [25]. More importantly, few drugs have been found to simultaneously regulate cholesterol efflux and endothelial cell inflammation, and further research in this field is expected to bring new breakthroughs in the treatment of atherosclerosis.

PPAR*γ* is a member of the ligand-activated receptor of the nuclear hormone receptor family. Human PPAR*γ* gene is located in the p25 region of chromosome 3 and contains 9 exons. Due to the different promoter selection and variable shearing mode, PPAR*γ* gene can be transcribed into a variety of mRNA, but only two proteins, PPAR*γ* 1 and PPAR*γ* 2, can be translated [26]. PPAR*γ* regulates glucose and lipid metabolism which was first used as a therapeutic target for diabetes. In recent years, more and more studies have found that PPAR*γ* plays an important role in protecting atherosclerosis. The basic action mode of PPAR*γ* is to form a heterodimer with RXR and activate transcription by binding to a specific DNA element called the peroxisome proliferation response element (PPRE) [27]. In addition, it can also participate in negative regulation of downstream molecules by recruiting corepressors or competitively binding coactivators of certain proteins. Previous studies have found that PPAR*γ* can affect the expression levels of cholesterol efflux and inflammatory reaction-related molecules through the above action modes [28, 29]. PPAR*γ* is widely distributed in a variety of cells in the vascular wall, including endothelial cells, macrophages, and smooth muscle cells [30]. Therefore, PPAR*γ* is considered to play a dual role in regulating macrophage cholesterol efflux and endothelial cell inflammation. In recent years, with the development of relevant studies, more and more evidence has shown that PPAR*γ* can enhance the cholesterol efflux efficiency through positively regulating PPAR*γ*/LXR*α*/ABCA1-ABCG1 pathway and can also inhibit endothelial cell inflammation via negatively regulating PPAR*γ*/NF-*κ*B p65 pathway, which is one of the important potential targets for the treatment of atherosclerosis [31, 32]. However, PPAR*γ* agonists have been found in clinical trials to increase the risk of water-sodium retention, osteoporosis, adipocyte hyperplasia, and myocardial hypertrophy, which greatly limits their applications in patients with cardiovascular disease [33, 34]. Interestingly, recent studies have found that natural compounds contained in Chinese herbs can selectively regulate PPAR*γ* with few side effects [35–37]. These results suggest that Chinese herbs and their active components may have unique advantages in regulating the activity of PPAR*γ*, which is expected to become a valuable complementary and alternative therapy.

In this study, we first identified 78 kinds of active components of QHZYF through UPLC-Q-TOF/MS analysis. One point to be noted here is that although water was used as the solvent in the preparation of QHZYF, the active ingredients contain the hydrophobic molecule linolenic acid. We reviewed the relevant literature and found that the similar phenomenon had also been reported in other study [38], which indicated that our preparation process and identification results are reliable. Future research may consider investigating the possible reason of this phenomenon. Then, the “drug-component-target” network diagram, PPI protein interaction diagram, and GO and KEGG enrichment analysis bubble charts were constructed by network pharmacology and bioinformatics analysis technology. Based on these results, we found that QHZYF might attenuate atherosclerosis via targeting PPAR*γ*-mediated PPAR*γ*/LXR*α*/ABCA1-ABCG1 and PPAR*γ*/NF-*κ*B p65 pathways to regulate cholesterol efflux and endothelial inflammation. Subsequently, ApoE^−/−^ mice were used to construct atherosclerosis model, and the efficacy of QHZYF was evaluated by oil red O staining, HE staining, Masson staining, Sirius red staining, and serum lipid detection. The results showed that QHZYF could significantly reduce the load of atherosclerotic plaque in aorta and the concentrations of TC and LDL-C in serum and increase the content of collagen fiber in plaque and the concentrations of HDL-C in serum. It is important to note that we have tried to use C57BL/6 wild-type mice to further verify the efficacy of QHZYF to eliminate the possible interference of apolipoprotein e gene knockout. The results showed that QHZYF also improved plaque load, collagen fiber content, and serum lipid content in wild-type mice to a certain extent. However, due to the small size of atherosclerotic plaques observed in our experiments, it was not possible to determine whether these plaques were nonspecific staining. Therefore, the results of the C57 mice should be interpreted with caution. Next, we conducted a preliminary study on the pharmacological mechanism of QHZYF in ApoE^−/−^ mice and C57 wild-type mice via immunohistochemistry, immunofluorescence, western blot, and ELISA. The results manifested that QHZYF could significantly increase the protein expression levels of PPAR*γ*, LXR*α*, ABCA1, and ABCG1 in the aorta of ApoE^−/−^ mice and wild-type mice and reduce the expression levels of NF-*κ*B p65, IL-1*β*, IL-6, and TNF-*α* in the aortic endothelial cells and reduce the concentrations of IL-1*β*, IL-6, and TNF-*α* in serum. These results suggested that the mechanism of QHZYF alleviating atherosclerosis may be related to cholesterol efflux and endothelial inflammation mediated by PPAR*γ*/LXR*α*/ABCA1-ABCG1 and PPAR*γ*/NF-*κ*B p65 pathways. Finally, we further verified these mechanisms in vitro. We used ox-LDL to intervene RAW 264.7 cells and HUVECs, respectively, to construct foam cell model and endothelial cell inflammation model in vitro. At the same time, they were treated with different concentrations of QHZYF. The results indicated that QHZYF could significantly reduce the lipid content and increase cholesterol efflux rate in RAW 264.7 cells, upregulate the protein and mRNA expression levels of PPAR*γ*, LXR*α*, ABCA1, and ABCG1, upregulate the protein and mRNA expression levels of PPAR*γ* in HUVECs, and downregulate the protein and mRNA expression levels of NF-*κ*B p65 and the mRNA expression levels of IL-1*β*, IL-6, and TNF-*α*, and reduce the concentrations of IL-1*β*, IL-6, and TNF-*α* in cell culture supernatant. In addition, after the PPAR*γ* gene was knocked down by the si-PPAR*γ* in the two kinds of cells, the above effects of QHZYF were significantly weakened. These results further confirmed that QHZYF attenuates atherosclerosis via targeting PPAR*γ*-mediated PPAR*γ*/LXR*α*/ABCA1-ABCG1 and PPAR*γ*/NF-*κ*B p65 pathways to regulate cholesterol efflux and endothelial cell inflammation.

This study systematically revealed the efficacy and potential mechanism of QHZYF in treating atherosclerosis by UPLC-Q-TOF/MS, network pharmacology, bioinformatics analysis, molecular docking, and experimental verification, providing new evidence for the role of TCM in atherosclerosis. More importantly, our study found for the first time that Chinese herbs could selectively regulate PPAR*γ* to play a multiple antiatherosclerosis role in enhancing cholesterol efflux efficiency and inhibiting endothelial cell inflammation, which is expected to make up for the limitations of current drug treatment methods and provide valuable ideas for future drug development. Further exploration of the role and pharmacological mechanism of Chinese herbs in the treatment of atherosclerosis will help to establish a more scientific and standardized prevention and treatment system that integrated TCM and modern medicine and give full play to the advantages of TCM. However, it should be acknowledged that this study has the following limitations: first, the results of network pharmacology and bioinformatics analysis showed that other targets and pathways may be involved in the treatment of atherosclerosis by QHZYF. However, due to the restriction of objective conditions, we failed to verify all the other targets and pathways in this study. Second, the pathogenesis of atherosclerosis involves the transcription, translation, and posttranslational modification of a variety of genes or proteins, as well as changes in various metabolic pathways, which cannot be fully covered by the research methods of network pharmacology. In the future, further research may consider revealing the pharmacological mechanism of QHZYF in treating atherosclerosis with the help of genomics, transcriptomics, proteomics, and metabolomics.

In conclusion, our study revealed that QHZYF could attenuate atherosclerosis via targeting PPAR*γ*-mediated PPAR*γ*/LXR*α*/ABCA1-ABCG1 and PPAR*γ*/NF-*κ*B p65 pathways to regulate cholesterol efflux and endothelial cell inflammatory response, providing direct and sufficient evidence for the treatment of atherosclerosis with TCM. More importantly, our study offers a promising complementary and alternative therapy which is expected to make up for the limitations of current drug treatment methods and provide a valuable reference for new drugs development in the future.

## Data Availability

The data used to support the findings of this study are included within the article and supplementary materials. For more detailed data, please contact the corresponding authors.

## Abbreviations

ABCA1: ATP-binding cassette transporter A1
ABCG1: ATP-binding cassette transporter G1
ApoE^−/−^: Apolipoprotein e-deficient
BSA: Bull serum albumin
CD31: Platelet endothelial cell adhesion molecule-1
Cy3: Cyanine 3
DAB: Diaminobenzene
DAPI: 4′,6-Diamidino2-phenylindole
HDL: High-density lipoprotein
HDL-C: High-density lipoprotein cholesterol
HMG-CoA: 3-Hydroxy-3-methylglutaryl-coenzyme A
HUVECs: Human umbilical vein endothelial cells
ICAM-1: Intercellular cell adhesion molecule-1
IL-1*β*: Interleukin-1*β*
IL-6: Interleukin-6
LDL-C: Low-density lipoprotein cholesterol
LXR*α*: Liver X receptor alpha
NF-*κ*B: Nuclear factor kappa-B
NPC1L1: Niemann-Pick C1-like 1
OCT: Optimal cutting temperature
ox-LDL: Oxidized low-density lipoprotein
PCSK9: Proprotein convertase subtilisin kexin type 9
PPAR*γ*: Peroxisome proliferator-activated receptor gamma
PVDF: Polyvinylidene fluoride
QHZYF: Qihuang Zhuyu Formula
RCT: Cholesterol reversal transport
TCM: Traditional Chinese medicine
TC: Total cholesterol
TG: Triglyceride
TNF-*α*: Tumor necrosis factor alpha
VCAM-1: Vascular endothelial cell adhesion molecule-1
ApoA-I: Apolipoprotein A-I
VLDL: Very low-density lipoprotein.

## Supplementary Materials

Supplementary Materials Supplementary 1. Supplementary Table S1: seventy-eight kinds of active components in QHZYF were identified by UPLC-Q-TOF/MS. Supplementary 2. Supplementary Table S2: the primer sequences used in RT-qPCR experiments.
