# Supplementary material for: Qihuang Zhuyu Formula Attenuates Atherosclerosis via Targeting PPARγ to Regulate Cholesterol Efflux and Endothelial Cell Inflammation
Source: Oxid Med Cell Longev. 2022 Dec 5;2022:2226168. doi: 10.1155/2022/2226168 (PMC9744610; doi:10.1155/2022/2226168)
Supplement: Supplementary Materials — Supplementary 1. Supplementary Table S1: seventy-eight kinds of active components in QHZYF were identified by UPLC-Q-TOF/MS. Supplementary 2. Supplementary Table S2: the primer sequences used in RT-qPCR experiments. [file 2226168.f1.docx]

**Supplementary Table S1**

| **Sequence number** | **Retention time** | **Ion mode** | **Components** | **Formula** | **mw** | **Calcd** | **Obsed** | **Error** | **MS/MS** | **Attribution** | |  |
| --- | --- | --- | --- | --- | --- | --- | --- | --- | --- | --- | --- | --- |
|  |  |  |  |  |  | ***m/z*** | ***m/z*** | **(ppm)** |  |  |  |  |
| 1 | 0.83 | [M-H]^-^ | Genipingentiobioside | C_23_H_34_O_15_ | 550.1898 | 549.1819 | 549.1827 | 1.46 | 385.1157[M-H-C_5_H_13_O_5_]^-^ | Leech | |  |
|  |  |  |  |  |  |  |  |  | 421.1081[M-H-C_3_H_13_O_5_]^-^ |  |  |  |
|  |  |  |  |  |  |  |  |  | 431.1165[M-H-C_5_H_11_O_3_]^-^ |  |  |  |
|  |  |  |  |  |  |  |  |  | 439.0854[M-H-C_4_H_15_O_3_]^-^ |  |  |  |
|  |  |  |  |  |  |  |  |  | 499.1754[M-H-H_3_O_3_]^-^ |  |  |  |
| 2 | 0.84 | [M-H]^-^ | D-mannoheptulose | C_7_H_14_O_7_ | 210.074 | 209.0661 | 209.0662 | 0.48 | 89.0288[M-H-C_4_H_9_O_4_]^-^ | Leech | |  |
|  |  |  |  |  |  |  |  |  | 119.0412[M-H-C_3_H_7_O_3_]^-^ |  |  |  |
|  |  |  |  |  |  |  |  |  | 143.0414[M-H-CH_7_O_3_]^-^ |  |  |  |
|  |  |  |  |  |  |  |  |  | 161.0465[M-H-CH_5_O_2_]^-^ |  |  |  |
|  |  |  |  |  |  |  |  |  | 179.0613[M-H-CCH_3_O]^-^ |  |  |  |
| 3 | 1 | [M+Cl]^-^ | 10-O-Methyl protosappanin b | C_17_H_18_O_6_ | 318.1103 | 353.0792 | 353.0764 | 7.93 | 133.0203[M-H-C_9_H_13_O_4_]^-^ | Sappan Wood | |  |
|  |  |  |  |  |  |  |  |  | 211.0046[M-H-C_5_H_15_O_2_]^-^ |  |  |  |
| 4 | 1.11 | [M-H]^-^ | Sucrose | C_12_H_22_O_11_ | 342.1162 | 341.1084 | 341.1086 | 0.59 | 341.1086[M-H]^-^ | Milkvetch Root | |  |
|  |  | [M+Cl]^-^ |  |  |  | 377.0851 | 377.0877 | 6.9 | 377.0877[M+Cl]^-^ |  |  |  |
| 5 | 1.3 | [M+CH_3_COO]^-^ | Bonducellin | C_17_H_14_O_4_ | 282.0892 | 341.1025 | 341.1057 | 9.38 | 341.1057[M+CH_3_COO]^-^ | Sappan Wood |  |  |
| 6 | 2.44 | [M-H]^-^ | Onjixanthone i | C_16_H_14_O_6_ | 302.079 | 301.0712 | 301.0736 | 7.97 | 271.0278[M-H-C_2_H_7_]^-^ | Safflower |  |  |
|  |  |  |  |  |  |  |  |  | 285.0463[M-H-CH_5_]^-^ |  |  |  |
| 7 | 2.53 | [M-H]^-^ | 1-Ribityl-2,3-diketo-1,2,3,4-tetrahydro-6,7-dimethyl-quinoxaline | C_15_H_20_N_2_O_6_ | 324.1321 | 323.1243 | 323.1272 | 8.98 | 323.1272[M-H]^-^ | Safflower |  |  |
| 8 | 2.7 | [M+CH_3_COO]^-^ | Brazilein | C_16_H_12_O_5_ | 284.0685 | 343.0818 | 343.0807 | 3.21 | 229.0530[M-H-C_3_H_3_O]^-^ | Sappan Wood |  |  |
| 9 | 2.92 | [M-H]- [M+HCOO]- | Hydroxysafflor yellow a | C_27_H_32_O_16_ | 612.169 | 611.1612 657.1667 | 611.1595 657.164 | 2.78 4.11 | 455.1067[M-H-C_4_H_13_O_6_]^-^ | Safflower |  |  |
|  |  |  |  |  |  |  |  |  | 461.1476[M-H-C_4_H_7_O_6_]^-^ |  |  |  |
|  |  |  |  |  |  |  |  |  | 463.0889[M-H-C_6_H_13_O_4_]^-^ |  |  |  |
|  |  |  |  |  |  |  |  |  | 473.1080[M-H-C_4_H_11_O_5_]^-^ |  |  |  |
|  |  |  |  |  |  |  |  |  | 491.1227[M-H-C_4_H_9_O_4_]^-^ |  |  |  |
| 10 | 2.94 | [M-H]^-^ | 6-Hydroxykaempferol-7-o-glucoside | C_21_H_20_O_12_ | 464.0955 | 463.0877 | 463.0868 | 1.94 | 257.0593[M-H-C_10_H_7_O_5_]^-^ | Safflower |  |  |
|  |  |  |  |  |  |  |  |  | 287.0676[M-H-C_9_H_5_O_4_]^-^ |  |  |  |
|  |  |  |  |  |  |  |  |  | 299.0480[M-H-C_9_H_9_O_3_]^-^ |  |  |  |
|  |  |  |  |  |  |  |  |  | 303.0800[M-H-C_9_H_5_O_3_]^-^ |  |  |  |
|  |  |  |  |  |  |  |  |  | 323.0427[M-H-C_7_H_9_O_3_]^-^ |  |  |  |
| 11 | 3.01 | [M-H]^-^ | Protosappanin a | C_15_H_12_O_5_ | 272.0685 | 271.606 | 271.6055 | 1.85 | 123.0483[M-H-C_8_H_5_O_3_]^-^ | Sappan Wood |  |  |
|  |  |  |  |  |  |  |  |  | 148.0200[M-H-C_7_H_8_O_2_]^-^ |  |  |  |
|  |  |  |  |  |  |  |  |  | 163.0450[M-H-C_6_H_5_O_2_]^-^ |  |  |  |
|  |  |  |  |  |  |  |  |  | 229.0538[M-H-C_2_H_3_O]^-^ |  |  |  |
| 12 | 3.18 | [M-H]^-^ | Protosappanin c | C_16_H_14_O_6_ | 302.079 | 301.0712 | 301.0736 | 7.97 | 227.0391[M-H-C_3_H_7_O_2_]^-^ | Sappan Wood |  |  |
|  |  |  |  |  |  |  |  |  |  |  |  | |
| 13 | 3.46 | [M-H]^-^ | Sappanchalcone | C_16_H_14_O_5_ | 286.0841 | 285.0763 | 285.0799 | 12.63 | 158.0382[M-H-C_6_H_8_O_3_]^-^ | Sappan Wood |  | |
|  |  |  |  |  |  |  |  |  | 161.0195[M-H-C_7_H_9_O_2_]^-^ |  |  | |
|  |  |  |  |  |  |  |  |  | 162.0358[M-H-C_7_H_8_O_2_]^-^ |  |  | |
|  |  |  |  |  |  |  |  |  | 163.0424[M-H-C_7_H_7_O_2_]^-^ |  |  | |
|  |  |  |  |  |  |  |  |  | 267.0712[M-H-H_3_O]^-^ |  |  | |
| 14 | 3.5 | [M+CH_3_COO]^-^ | 3,4-Dihydroxyrottlerin | C_30_H_28_O_10_ | 548.1682 | 607.1816 | 607.1795 | 3.46 | 227.0367[M-H-C_17_H_21_O_6_]^-^ | Safflower |  | |
|  |  |  |  |  |  |  |  |  | 229.0546[M-H-C_17_H_19_O_6_]^-^ |  |  | |
|  |  |  |  |  |  |  |  |  | 232.0729[M-H-C_17_H_16_O_6_]^-^ |  |  | |
|  |  |  |  |  |  |  |  |  | 241.0557[M-H-C_16_H_19_O_6_]^-^ |  |  | |
|  |  |  |  |  |  |  |  |  | 243.0689[M-H-C_16_H_17_O_6_]^-^ |  |  | |
| 15 | 3.5 | [M-H]^-^ | Episappanol | C_16_H_16_O_6_ | 304.0947 | 303.0869 | 303.0864 | 7.59 | 109.0325[M-H-C_10_H_11_]^-^ | Sappan Wood |  | |
|  |  |  |  |  |  |  |  |  | 143.0471[M-H-C_6_H_9_O_5_]^-^ |  |  | |
|  |  |  |  |  |  |  |  |  | 158.0382[M-H-C_6_H_10_O_4_]^-^ |  |  | |
|  |  |  |  |  |  |  |  |  | 159.0461[M-H-C_6_H_9_O_4_]^-^ |  |  | |
|  |  |  |  |  |  |  |  |  | 255.0608[M-H-CH_5_O_2_]^-^ |  |  | |
| 16 | 3.65 | [M-H]^-^ | Geniposide | C_17_H_24_O_10_ | 388.1369 | 387.1291 | 387.1249 | 10.85 | 257.0351[M-H-C_7_H_15_O_2_]^-^ | Leech |  | |
|  |  |  |  |  |  |  |  |  | 271.0319[M-H-C_3_H_17_O_4_]^-^ |  |  | |
|  |  |  |  |  |  |  |  |  | 286.0493[M-H-C_2_H_14_O_4_]^-^ |  |  | |
|  |  |  |  |  |  |  |  |  | 287.0598[M-H-C_2_H_13_O_4_]^-^ |  |  | |
|  |  |  |  |  |  |  |  |  | 301.0576[M-H-C_5_H_11_O]^-^ |  |  | |
| 17 | 3.82 | [M-H]^-^ | Neocarthamin | C_21_H_22_O_11_ | 450.1162 | 449.1084 | 449.1064 | 4.45 | 257.0351[M-H-C_11_H_13_O_3_]^-^ | Safflower |  | |
|  |  |  |  |  |  |  |  |  | 271.0319[M-H-C_7_H_15_O_5_]^-^ |  |  | |
|  |  |  |  |  |  |  |  |  | 286.0493[M-H-C_6_H_12_O_5_]^-^ |  |  | |
|  |  |  |  |  |  |  |  |  | 287.0598[M-H-C_6_H_11_O_5_]^-^ |  |  | |
| 18 | 3.85 | [M+HCOO]^-^ | Vitexin xyloside | C_26_H_28_O_15_ | 580.1428 | 625.1405 | 625.1428 | 3.68 | 288.0838[M-H-C_14_H_12_O_7_]^-^ | Manyflower So1omonseal Rhizome |  | |
|  |  |  |  |  |  |  |  |  | 299.0249[M-H-C_11_H_21_O_8_]^-^ |  |  | |
|  |  |  |  |  |  |  |  |  | 344.0755[M-H-C_12_H_12_O_5_]^-^ |  |  | |
|  |  |  |  |  |  |  |  |  | 358.0714[M-H-C_6_H_14_O_7_]^-^ |  |  | |
|  |  |  |  |  |  |  |  |  | 463.1181[M-H-C_8_H_5_O]^-^ |  |  | |
| 19 | 3.94 | [M+CH_3_COO]^-^ | Uridine | C_9_H_12_N_2_O_6_ | 244.0695 | 303.0828 | 303.0864 | 11.88 | 303.0864[M+CH_3_COO]^-^ | Milkvetch Root |  | |
| 20 | 3.98 | [M+Cl]^-^ | 2'-Hydroxy-3',4'-dime thoxy-isoflavane-7-o-β-d-glucoside | C_23_H_32_O_10_ | 468.1995 | 503.1684 | 503.1718 | 6.76 | 503.1718[M+Cl]^-^ | Milkvetch Root |  | |
| 21 | 4.02 | [M-H]^-^ | Saffloryellow | C_27_H_30_O_16_ | 610.1534 | 609.1456 | 609.1409 | 7.72 | 284.0312[M-H-C_12_H_22_O_10_]^-^ | Safflower |  | |
|  |  |  |  |  |  |  |  |  | 285.0361[M-H-C_12_H_21_O_10_]^-^ |  |  | |
|  |  |  |  |  |  |  |  |  | 447.0869[M-H-C_6_H_11_O_5_]^-^ |  |  | |
|  |  |  |  |  |  |  |  |  | 448.0734[M-H-C_7_H_14_O_4_]^-^ |  |  | |
| 22 | 4.08 | [M-H]^-^ | Safflomin a | C_27_H_32_O_16_ | 612.169 | 611.1612 | 611.1595 | 2.78 | 611.1595[M-H]^-^ | Safflower |  | |
| 23 | 4.19 | [M-H]^-^ | 3'-Deoxysappanol | C_16_H_16_O_5_ | 288.0998 | 287.0919 | 287.0904 | 5.23 | 137.0300[M-H-C_9_H_11_O_2_]^-^ | Sappan Wood |  | |
| 24 | 4.27 | [M+HCOO]^-^ | Gardenoside | C_17_H_24_O_11_ | 404.1319 | 449.1295 | 449.125 | 10.02 | 119.0470[M-H-C_9_H_14_O_10_]^-^ | Leech |  | |
|  |  |  |  |  |  |  |  |  | 121.0295[M-H-C_10_H_19_O_9_]^-^ |  |  | |
| 25 | 4.4 | [M-H]^-^ | Brasilin | C_16_H_14_O_5_ | 286.0841 | 285.0763 | 285.0725 | 13.33 | 227.0355[M-H-C_3_H_7_O]^-^ | Sappan Wood |  | |
| 26 | 4.4 | [M-H]^-^ | Safflower yellow a | C_27_H_30_O_15_ | 594.1585 | 593.1506 | 593.1549 | 7.25 | 119.0492[M-H-C_19_H_23_O_14_]^-^ | Safflower |  | |
|  |  |  |  |  |  |  |  |  | 284.0279[M-H-C_12_H_22_O_9_]^-^ |  |  | |
|  |  |  |  |  |  |  |  |  | 449.1014[M-H-C_6_H_9_O_4_]^-^ |  |  | |
| 27 | 4.46 | [M-H]^-^ | 3'-Hydroxy-4--methoxyisoflavone-7-o-beta-d-glucoside | C_22_H_22_O_10_ | 446.1213 | 445.1135 | 445.1145 | 2.25 | 160.0422[M-H-C_16_H_14_O_5_]^-^ | Milkvetch Root |  | |
|  |  |  |  |  |  |  |  |  | 161.0249[M-H-C_3_H_17_O_7_]^-^ |  |  | |
|  |  |  |  |  |  |  |  |  | 163.0385[M-H-C_13_H_15_O_7_]^-^ |  |  | |
|  |  |  |  |  |  |  |  |  | 223.0700[M-H-C_11_H_11_O_5_]^-^ |  |  | |
|  |  |  |  |  |  |  |  |  | 267.0662[M-H-C_6_H_11_O_6_]^-^ |  |  | |
| 28 | 4.65 | [M-H]^-^ | Protosappanin a Dimethyl acetal | C_17_H_18_O_6_ | 318.1103 | 317.1025 | 317.1068 | 13.56 | 227.0350[M-H-C_4_H_11_O_2_]^-^ | Sappan Wood |  | |
|  |  |  |  |  |  |  |  |  | 229.0506[M-H-C_4_H_9_O_2_]^-^ |  |  | |
| 29 | 4.73 | [M-H]^-^ | Calycosin | C_16_H_12_O_5_ | 284.0685 | 283.0606 | 283.0617 | 3.89 | 148.0116[M-H-C_8_H_8_O_2_]^-^ | Milkvetch Root |  | |
|  |  |  |  |  |  |  |  |  | 268.0334[M-H-CH_4_]^-^ |  |  | |
| 30 | 4.92 | [M-H]^-^ | Mannose | C_6_H_12_O_6_ | 180.0634 | 179.0556 | 179.0537 | 10.61 | 179.0537[M-H]^-^ | Manyflower So1omonseal Rhizome |  | |
| 31 | 5.25 | [M-H]^-^ | 3,7-Dihydroxychroman-4-one | C_9_H_8_O_4_ | 180.0423 | 179.0344 | 179.0341 | 1.68 | 151.0402[M-H-CHO]^-^ | Sappan Wood |  | |
| 32 | 5.72 | [M-H]^-^ | 3'-Methoxy-4',5,7-trihydroxyflavone | C_16_H_12_O_6_ | 300.0634 | 299.0566 | 299.0599 | 13.29 | 269.0447[M-H-CH_3_O]^-^ | Sappan Wood |  | |
| 33 | 5.73 | [M+CH_3_COO]^-^ | Carthamone | C_21_H_20_O_11_ | 448.1006 | 507.1139 | 507.1106 | 6.51 | 269.0447[M-H-C_6_H_11_O_6_]^-^ | Safflower |  | |
|  |  |  |  |  |  |  |  |  | 364.0685[M-H-CH_8_O_4_]^-^ |  |  | |
| 34 | 5.88 | [M-H]^-^ | Cartormin | C_27_H_29_NO_13_ | 575.1639 | 574.1561 | 574.1585 | 4.18 | 283.0528[M-H-C_12_H_20_O_8_]^-^ | Safflower |  | |
| 35 | 6.22 | [M+HCOO]^-^ | Benzothiazole | C_14_H_12_N_2_S | 240.0721 | 285.0698 | 285.0722 | 8.42 | 285.0722[M+HCOO]^-^ | Safflower |  | |
| 36 | 7.6 | [M+HCOO]^-^ | Dibenzoxocin | C_15_H_12_O_2_ | 224.0837 | 269.0814 | 269.0842 | 10.41 | 269.0842[M+HCOO]^-^ | Sappan Wood |  | |
| 37 | 7.67 | [M-H]^-^ | 5,6-Dihydroxy-7-o-glucoside-flavone | C_21_H_20_O_10_ | 432.1056 | 431.0978 | 431.0926 | 10.2 | 269.0427[M-H-C_6_H_11_O_5_]^-^ | Manyflower So1omonseal Rhizome |  | |
| 38 | 8.73 | [M+Cl]^-^ | Sagittariol | C_19_H_32_O_2_ | 292.2402 | 327.2091 | 327.2092 | 0.31 | 327.2092[M+Cl]^-^ | Safflower |  | |
| 39 | 11.23 | [M+HCOO]^-^ | Astragaloside II | C_43_H_70_O_15_ | 826.4715 | 871.4691 | 871.4696 | 0.57 | 871.4696[M+HCOO]^-^ | Milkvetch Root |  | |
| 40 | 12.58 | [M+HCOO]^-^ | Astragaloside I | C_45_H_72_O_16_ | 868.482 | 913.4797 | 913.4788 | 0.99 | 913.4788[M+HCOO]^-^ | Milkvetch Root |  | |
| 41 | 12.58 | [M+Cl]^-^ | Astragaloside V | C_47_H_78_O_19_ | 946.5137 | 981.4826 | 981.4795 | 3.16 | 913.5249[M-H-HO_2_]^-^ | Milkvetch Root |  | |
|  |  |  |  |  |  |  |  |  | 914.4943[M-H-CH_4_O]^-^ |  |  | |
| 42 | 28.29 | [M+HCOO]^-^ | (z, e, e)-1,3,5,11-Tridecatetraene-7,9-diyne | C_13_H_12_ | 168.0939 | 213.0916 | 213.0931 | 7.04 | 213.0931[M+HCOO]^-^ | Safflower |  | |
| 43 | 28.3 | [M+CH_3_COO]^-^ | (z,z)-1,3,11-Tridecatriene-5,7,9-triyne | C_13_H_10_ | 166.0783 | 225.0916 | 225.094 | 10.67 | 225.0940[M+CH_3_COO]^-^ | Safflower |  | |
| 44 | 28.34 | [M+HCOO]^-^ | (-)-Methyl selina-3,11-dien-14-oate | C_16_H_24_O_2_ | 248.1776 | 293.1753 | 293.1718 | 11.95 | 92.9982[M-H-C_11_H_23_]^-^ | Sappan Wood |  | |
|  |  |  |  |  |  |  |  |  | 119.0502[M-H-C_8_H_14_O]^-^ |  |  | |
|  |  |  |  |  |  |  |  |  | 121.0659[M-H-C_8_H_15_O]^-^ |  |  | |
|  |  |  |  |  |  |  |  |  |  |  |  | |
|  |  |  |  |  |  |  |  |  |  |  |  | |
| 45 | 2.51 | [M+H]^+^ | 6-Hydroxykaempferol-7-o-glucoside | C_21_H_20_O_12_ | 464.0955 | 465.1033 | 465.1043 | 2.15 | 115.0466[M+H-C_16_H_13_O_9_]^+^ | Safflower |  | |
|  |  |  |  |  |  |  |  |  | 303.0478[M+H-C_6_H_9_O_5_]^+^ |  |  | |
|  |  |  |  |  |  |  |  |  | 319.0410[M+H-C_6_H_9_O_4_]^+^ |  |  | |
| 46 | 2.92 | [M+H]^+^ | 5,6-Dihydroxy-7-o-glucoside-flavone | C_21_H_20_O_10_ | 432.1056 | 433.1135 | 433.1136 | 0.23 | 301.0650[M+H-C_9_H_7_O]^+^ | Manyflower So1omonseal Rhizome |  | |
|  |  |  |  |  |  |  |  |  | 303.0499[M+H-C_6_H_9_O_3_]^+^ |  |  | |
|  |  |  |  |  |  |  |  |  | 313.0701[M+H-C_4_H_7_O_4_]^+^ |  |  | |
|  |  |  |  |  |  |  |  |  | 314.0578[M+H-C_8_H_6_O]^+^ |  |  | |
|  |  |  |  |  |  |  |  |  | 331.0861[M+H-C_4_H_5_O_3_]^+^ |  |  | |
| 47 | 2.92 | [M+H]^+^ | Neocarthamin | C_21_H_22_O_11_ | 450.1162 | 451.124 | 451.1217 | 5.01 | 303.0468[M+H-C_6_H_11_O_4_]^+^ | Safflower |  | |
|  |  |  |  |  |  |  |  |  | 313.0705[M+H-C_4_H_9_O_4_]^+^ |  |  | |
|  |  |  |  |  |  |  |  |  | 314.0578[M+H-C_8_H_8_O_2_]^+^ |  |  | |
|  |  |  |  |  |  |  |  |  | 331.0861[M+H-C_4_H_7_O_4_]^+^ |  |  | |
|  |  |  |  |  |  |  |  |  | 415.0991[M+H-H_3_O_2_]^+^ |  |  | |
| 48 | 2.92 | [M+Na]^+^ | Neosappanone a | C_33_H_28_O_11_ | 600.1632 | 623.1529 | 623.1545 | 2.57 | 91.0478[M+H-C_30_H_21_O_7_]^+^ | Sappan Wood |  | |
|  |  |  |  |  |  |  |  |  | 97.0239[M+H-C_28_H_23_O_8_]^+^ |  |  | |
|  |  |  |  |  |  |  |  |  | 121.0223[M+H-C_26_H_23_O_8_]^+^ |  |  | |
|  |  |  |  |  |  |  |  |  | 289.0664[M+H-C_18_H_15_O_5_]^+^ |  |  | |
|  |  |  |  |  |  |  |  |  | 301.0650[M+H-C_17_H_15_O_5_]^+^ |  |  | |
| 49 | 2.92 | [M+H]^+^ | Ombuin | C_17_H_14_O_7_ | 330.074 | 331.0818 | 331.0818 | 0 | 147.0382[M+H-C_8_H_7_O_5_]^+^ | Sappan Wood |  | |
|  |  |  |  |  |  |  |  |  | 193.0050[M+H-C_8_H_9_O_2_]^+^ |  |  | |
|  |  |  |  |  |  |  |  |  | 207.0265[M+H-C_7_H_7_O_2_]^+^ |  |  | |
|  |  |  |  |  |  |  |  |  | 301.0650[M+H-CHO]^+^ |  |  | |
|  |  |  |  |  |  |  |  |  | 303.0468[M+H-C_2_H_3_]^+^ |  |  | |
| 50 | 2.92 | [M+K]^+^ | Safflomin a | C_27_H_32_O_16_ | 612.169 | 651.1327 | 651.1367 | 6.14 | 651.1367[M+K]^+^ | Safflower |  | |
| 51 | 3.18 | [M+H]^+^ | Calycosin | C_16_H_12_O_5_ | 284.0685 | 285.0763 | 285.0737 | 9.12 | 285.0737[M+H]^+^ | Milkvetch Root |  | |
| 52 | 3.18 | [M+H]^+^ | Onjixanthone i | C_16_H_14_O_6_ | 302.079 | 303.0869 | 303.0885 | 5.28 | 287.0563[M+H-CH_3_]^+^ | Safflower |  | |
| 53 | 3.33 | [M+Na]^+^ | 1-Ribityl-2,3-diketo-1,2,3,4-tetrahydro-6,7-dimethyl-quinoxaline | C_15_H_20_N_2_O_6_ | 324.1321 | 347.1219 | 347.1227 | 2.31 | 144.0735[M+H-C_6_H_12_O_6_]^+^ | Safflower |  | |
| 54 | 3.48 | [M+H]^+^ | Brazilein | C_16_H_12_O_5_ | 284.0685 | 285.0763 | 285.0737 | 9.12 | 229.0465[M+H-C_3_H_3_O]^+^ | Sappan Wood |  | |
|  |  |  |  |  |  |  |  |  | 237.0545[M+H-CH_3_O_2_]^+^ |  |  | |
|  |  |  |  |  |  |  |  |  | 239.0651[M+H-CHO_2_]^+^ |  |  | |
|  |  |  |  |  |  |  |  |  | 251.0710[M+H-HO_2_]^+^ |  |  | |
|  |  |  |  |  |  |  |  |  | 267.0585[M+H-HO]^+^ |  |  | |
| 55 | 3.5 | [M+K]^+^ | Episappanol | C_16_H_16_O_6_ | 304.0947 | 343.0584 | 343.0587 | 0.87 | 147.0371[M+H-C_7_H_9_O_4_]^+^ | Sappan Wood |  | |
|  |  |  |  |  |  |  |  |  | 165.0626[M+H-C_7_H_7_O_3_]^+^ |  |  | |
|  |  |  |  |  |  |  |  |  | 183.0731[M+H-C_7_H_5_O_2_]^+^ |  |  | |
|  |  |  |  |  |  |  |  |  | 195.0730[M+H-C_6_H_5_O_2_]^+^ |  |  | |
|  |  |  |  |  |  |  |  |  | 251.0710[M+H-H_5_O_3_]^+^ |  |  | |
| 56 | 3.5 | [M+NH_4_]^+^ | Methyl brevifolin carboxylate | C_14_H_10_O_8_ | 306.0376 | 324.0719 | 324.0717 | 0.62 | 139.0449[M+H-C_7_H_3_O_5_]^+^ | Sappan Wood |  | |
|  |  |  |  |  |  |  |  |  | 141.0615[M+H-C_7_HO_5_]^+^ |  |  | |
|  |  |  |  |  |  |  |  |  | 155.0425[M+H-C_7_H_3_O_4_]^+^ |  |  | |
|  |  |  |  |  |  |  |  |  | 185.0518[M+H-C_6_HO_3_]^+^ |  |  | |
|  |  |  |  |  |  |  |  |  | 205.0573[M+H-C_3_HO_4_]^+^ |  |  | |
| 57 | 3.61 | [M+H]^+^ | Genipingentiobioside | C_23_H_34_O_15_ | 550.1898 | 551.1976 | 551.196 | 2.9 | 551.1960[M+H]^+^ | Leech |  | |
| 58 | 3.82 | [M+H]^+^ | Safflower yellow A | C_27_H_30_O_15_ | 594.1585 | 595.1663 | 595.1693 | 5.04 | 181.0077[M+H-C_19_H_25_O_10_]^+^ | Safflower |  | |
|  |  |  |  |  |  |  |  |  | 247.0258[M+H-C_15_H_23_O_9_]^+^ |  |  | |
|  |  |  |  |  |  |  |  |  | 289.0774[M+H-C_12_H_17_O_9_]^+^ |  |  | |
|  |  |  |  |  |  |  |  |  | 303.0442[M+H-C_12_H_19_O_8_]^+^ |  |  | |
|  |  |  |  |  |  |  |  |  | 433.1174[M+H-C_6_H_9_O_5_]^+^ |  |  | |
| 59 | 3.83 | [M+NH_4_]^+^ | Aflatoxing1 | C_17_H_12_O_7_ | 328.0583 | 346.0927 | 346.0902 | 7.23 | 229.0517[M+H-C_4_H_3_O_3_]^+^ | Leech |  | |
|  |  |  |  |  |  |  |  |  | 247.0258[M+H-C_5_H_5_O]^+^ |  |  | |
|  |  |  |  |  |  |  |  |  | 259.0201[M+H-C_4_H_5_O]^+^ |  |  | |
|  |  |  |  |  |  |  |  |  | 303.0442[M+H-C_2_H]^+^ |  |  | |
| 60 | 3.83 | [M+H]^+^ | Carthamidin | C_15_H_12_O_6_ | 288.0634 | 289.0712 | 289.0763 | 8.3 | 119.0421[M+H-C_7_H_5_O_5_]^+^ | Safflower |  | |
|  |  |  |  |  |  |  |  |  | 169.0054[M+H-C_8_H_7_O]^+^ |  |  | |
| 61 | 3.85 | [M+NH_4_]^+^ | Saffloryellow | C_27_H_30_O_16_ | 610.1534 | 628.1878 | 628.1808 | 11.12 | 259.0201[M+H-C_14_H_23_O_10_]^+^ | Safflower |  | |
|  |  |  |  |  |  |  |  |  | 289.0774[M+H-C_12_H_17_O_10_]^+^ |  |  | |
|  |  |  |  |  |  |  |  |  | 303.0442[M+H-C_12_H_19_O_9_]^+^ |  |  | |
|  |  |  |  |  |  |  |  |  | 327.0184[M+H-C_11_H_23_O_8_]^+^ |  |  | |
|  |  |  |  |  |  |  |  |  | 433.1174[M+H-C_6_H_9_O_6_]^+^ |  |  | |
| 62 | 4.02 | [M+H]^+^ | Kaempferol | C_15_H_10_O_6_ | 286.0477 | 287.0556 | 287.0531 | 8.71 | 287.0531[M+H]^+^ | Milkvetch Root and Safflower |  | |
| 63 | 4.35 | [M+Na]^+^ | Uridine | C_9_H_12_N_2_O_6_ | 244.0695 | 267.0593 | 267.0612 | 7.12 | 72.0375[M+H-C_6_H_6_NO_5_]^+^ | Milkvetch Root |  | |
|  |  |  |  |  |  |  |  |  | 181.0591[M+H-CH_3_O_3_]^+^ |  |  | |
|  |  |  |  |  |  |  |  |  | 195.0760[M+H-HO_3_]^+^ |  |  | |
|  |  |  |  |  |  |  |  |  | 197.0489[M+H-CH_3_O_2_]^+^ |  |  | |
|  |  |  |  |  |  |  |  |  | 211.0716[M+H-HO_2_]^+^ |  |  | |
| 64 | 4.5 | [M+NH_4_]^+^ | Suffruticoside A | C_27_H_32_O_16_ | 612.169 | 630.2034 | 630.1962 | 11.43 | 165.0630[M+H-C_18_H_23_O_13_]^+^ | Milkvetch Root |  | |
| 65 | 4.73 | [M+H]^+^ | 3'-Hydroxy-4--methoxyisoflavone-7-o-beta-d-glucoside | C_22_H_22_O_10_ | 446.1213 | 447.1291 | 447.1251 | 8.95 | 169.0566[M+H-C_14_H_13_O_6_]^+^ | Milkvetch Root |  | |
|  |  |  |  |  |  |  |  |  | 196.0451[M+H-C_13_H_14_O_5_]^+^ |  |  | |
|  |  |  |  |  |  |  |  |  | 197.0510[M+H-C_13_H_13_O_5_]^+^ |  |  | |
|  |  |  |  |  |  |  |  |  | 253.0407[M+H-C_7_H_13_O_6_]^+^ |  |  | |
|  |  |  |  |  |  |  |  |  | 270.0465[M+H-C_7_H_12_O_5_]^+^ |  |  | |
| 66 | 4.88 | [M+NH_4_]^+^ | Cartormin | C_27_H_29_NO_13_ | 575.1639 | 593.1983 | 593.1985 | 0.34 | 147.0388[M+H-C_18_H_22_NO_11_]^+^ | Safflower |  | |
|  |  |  |  |  |  |  |  |  | 303.0353[M+H-C_13_H_20_O_6_]^+^ |  |  | |
|  |  |  |  |  |  |  |  |  | 313.0820[M+H-C_14_H_14_O_5_]^+^ |  |  | |
| 67 | 5.55 | [M+H]^+^ | Isorhamnetin | C_16_H_12_O_7_ | 316.0583 | 317.0661 | 317.0667 | 1.89 | 317.0667[M+H]^+^ | Milkvetch Root |  | |
| 68 | 5.6 | [M+H]^+^ | Protosappanin A | C_15_H_12_O_5_ | 272.0685 | 273.0763 | 273.0746 | 6.23 | 273.0746[M+H]^+^ | Sappan Wood |  | |
| 69 | 5.73 | [M+H]^+^ | Rhamnocitrin | C_16_H_12_O_6_ | 300.0634 | 301.0712 | 301.0742 | 9.97 | 153.0131[M+H-C_9_H_7_O_2_]^+^ | Milkvetch Root |  | |
|  |  |  |  |  |  |  |  |  | 229.0495[M+H-C_3_H_3_O_2_]^+^ |  |  | |
|  |  |  |  |  |  |  |  |  | 271.0601[M+H-CHO]^+^ |  |  | |
| 70 | 6.05 | [M+H]^+^ | Sappanone B | C_16_H_14_O_6_ | 302.079 | 303.0869 | 303.0885 | 5.28 | 269.0763[M+H-HO_2_]^+^ | Sappan Wood |  | |
| 71 | 7.44 | [M+Na]^+^ | 6-Dimethoxy-isoflavane | C_23_H_28_O_10_ | 464.1682 | 487.158 | 487.1608 | 5.74 | 487.1608[M+Na]^+^ | Milkvetch Root |  | |
| 72 | 9.04 | [M+K]^+^ | (z, e, e)-1,3,5,11-Tridecatetraene-7,9-diyne | C_13_H_12_ | 168.0939 | 207.0576 | 207.0585 | 4.35 | 207.0585[M+K]^+^ | Safflower |  | |
| 73 | 9.52 | [M+H]^+^ | Formononetin | C_16_H_12_O_4_ | 268.0736 | 269.0814 | 269.0845 | 11.52 | 237.0589[M+H-CH_3_O]^+^ | Milkvetch Root |  | |
|  |  | [M+Na]^+^ |  |  |  |  |  |  | 253.0495[M+H-CH_3_]^+^ |  |  | |
| 74 | 12.11 | [M+Na]^+^ | Astragaloside I | C_45_H_72_O_16_ | 868.482 | 891.4718 | 891.4687 | 3.48 | 119.0736[M+H-C_40_H_61_O_13_]^+^ | Milkvetch Root |  | |
|  |  |  |  |  |  |  |  |  | 711.3990[M+H-C_8_H_13_O_3_]^+^ |  |  | |
|  |  |  |  |  |  |  |  |  | 775.4300[M+H-C_3_H_9_O_3_]^+^ |  |  | |
| 75 | 14.82 | [M+H]^+^ | Linolenicacid | C_18_H_30_O_2_ | 278.2246 | 279.2324 | 279.2353 | 10.39 | 279.2353[M+H]^+^ | Safflower |  | |
| 76 | 15.82 | [M+NH_4_]^+^ | Genioisidic acid | C_16_H_22_O_10_ | 374.1213 | 392.1557 | 392.151 | 11.99 | 392.1510[M+NH_4_]^+^ |  |  | |
| 77 | 21.54 | [M+NH_4_]^+^ | N-Candicine | C_11_H_18_NO | 180.1388 | 198.1732 | 198.1748 | 8.08 | 91.0446[M+H-C_4_H_11_NO]^+^ | Milkvetch Root |  | |
|  |  |  |  |  |  |  |  |  | 93.0616[M+H-C_4_H_9_NO]^+^ |  |  | |
|  |  |  |  |  |  |  |  |  | 107.0765[M+H-C_3_H_7_NO]^+^ |  |  | |
|  |  |  |  |  |  |  |  |  | 139.1009[M+H-C_3_H_5_]^+^ |  |  | |
|  |  |  |  |  |  |  |  |  | 149.1225[M+H-CH_3_O]^+^ |  |  | |
| 78 | 28.36 | [M+Na]^+^ | Sucrose | C_12_H_22_O_11_ | 342.1162 | 365.106 | 365.1099 | 10.68 | 365.1099[M+Na]^+^ | Milkvetch Root |  | |

**Supplementary Table S2**

| **Name** | **Species** | **Forward primer** | **Reverse primer** |
| --- | --- | --- | --- |
| PPARγ | Human | GGGATCAGCTCCGTGGATCT | TGCACTTTGGTACTCTTGAAGTT |
| NF-κB p65 | Human | ATGTGGAGATCATTGAGCAGC | CCTGGTCCTGTGTAGCCATT |
| IL-1β | Human | ATGATGGCTTATTACAGTGGCAA | GTCGGAGATTCGTAGCTGGA |
| IL-6 | Human | ACTCACCTCTTCAGAACGAATTG | CCATCTTTGGAAGGTTCAGGTTG |
| TNF-α | Human | GAGGCCAAGCCCTGGTATG | CGGGCCGATTGATCTCAGC |
| GAPDH | Human | GGACCTGACCTGCCGTCTAG | GTAGCCCAGGATGCCCTTGA |
| PPARγ | Mouse | GGAAGACCACTCGCATTCCTT | GTAATCAGCAACCATTGGGTCA |
| LXRα | Mouse | ACAGAGCTTCGTCCACAAAAG | GCGTGCTCCCTTGATGACA |
| ABCA1 | Mouse | GCTTGTTGGCCTCAGTTAAGG | GTAGCTCAGGCGTACAGAGAT |
| ABCG1 | Mouse | GTGGATGAGGTTGAGACAGACC | CCTCGGGTACAGAGTAGGAAAG |
| GAPDH | Mouse | AAGAAGGTGGTGAAGCAGG | GAAGGTGGAAGAGTGGGAGT |
